# Nano‐Zinc Sulfide Modified 3D Reconstructed Zinc Anode with Induced Deposition Effect Assists Long‐Cycle Stable Aqueous Zinc Ion Battery

**DOI:** 10.1002/advs.202417323

**Published:** 2025-01-21

**Authors:** Dongfang Guo, Fengyu Li, Bin Zhang

**Affiliations:** ^1^ School of Physics and Microelectronics Zhengzhou University Zhengzhou 450001 China; ^2^ School of Physics and Laboratory of Zhongyuan Light Zhengzhou University Zhengzhou 450001 China

**Keywords:** anode, aqueous batteries, dendritic‐free, zinc ion

## Abstract

Aqueous zinc ion batteries are often adversely affected by the poor stability of zinc metal anodes. Persistent water‐induced side reactions and uncontrolled dendrite growth have seriously damaged the long‐term service life of aqueous zinc ion batteries. In this paper, it is reported that a zinc sulfide with optimized electron arrangement on the surface of zinc anode is used to modify the zinc anode to achieve long‐term cycle stability of zinc anode. The effective active sites of the zinc metal anode surface are first significantly improved by a simple ultrasound‐assisted etching strategy, and then the in situ zinc sulfide interface phase further guides the zinc ion deposition behavior on the surface of the zinc metal anode. The zinc sulfide protective layer well regulates the interfacial electric field and the migration of Zn^2+^, thereby significantly promoting the homogenization of zinc ion flux to achieve dendrite‐free deposition. In addition, the aqueous zinc ion full cell assembled based on ZnS@3D‐Zn anode achieves better output performance in long‐term cycles. In summary, this work sheds light on the importance of reasonable interfacial modification for the development of dendrite‐free and stable zinc anode chemistry, which opens up a new path for promoting the development of zinc‐based batteries.

## Graphical abstract

A 3D reconstructed zinc anode based on zinc sulfide nanolayer modification is used as an anode for aqueous zinc ion batteries. The 3D structure provides a large number of effective active sites for the zinc deposition process. The in situ ZnS interface phase further regulates the interfacial electric field and the ordered deposition of Zn^2+^ while inhibiting the anode side reaction.

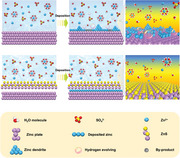

## 1 Introduction

With the rapid popularization of portable electronic products and electric vehicles, it is urgent to develop sustainable clean power sources with both cost‐effectiveness and high safety.^[^ ^1^ ^]^ Rechargeable aqueous zinc ion batteries are considered to be a promising candidate technology for grid energy storage applications due to low cost, high safety, environmental friendliness, excellent theoretical gravimetric/volumetric capacity (820 mAh g^−1^ and 5855 mAh cm^−3^), and suitable zinc anode redox potential (−0.76 V vs standard hydrogen electrode).^[^ ^2^ ^]^ Unfortunately, the zinc metal interface in weak acidic electrolyte conditions will expose some inherent defects, such as disordered dendrite growth, hydrogen evolution reaction, water‐induced by‐product formation, and corrosion during electrochemical reactions. These adverse behaviors seriously damage the long cycle life of aqueous zinc ion batteries, especially at high current densities. The inhomogeneity of the electric field distribution and the poor electrolyte concentration at high current density will aggravate the disordered zinc deposition, which will produce dendrites and eventually lead to short circuit of the battery.^[^ ^3^ ^]^ In addition, the continuous accumulation of a series of harmful side reactions is extremely unfavorable to the rapid electrochemical kinetics and thermodynamic stability of the electrode, which will greatly reduce the redox reversibility and effective utilization of the zinc anode.^[^ ^4^ ^]^ Therefore, optimizing the long‐term interface stability of zinc metal anodes is an inevitable choice to promote the commercialization of aqueous zinc ion batteries. Benefiting from the special solvation structure of zinc ions in an aqueous solution, the occurrence of water‐induced side reactions can be fundamentally alleviated by reducing the water content in the electrolyte.^[^ ^5^ ^]^ The high‐concentration “salt‐in‐water” electrolyte based on this purpose is to adjust the electrochemical behavior of cations by effectively reducing the dominant position of H_2_O in the inner solvation structure.^[^ ^6^ ^]^ It cannot be ignored that the high‐cost “salt‐in‐water” electrolyte is extremely difficult for the vigorous promotion of zinc ion batteries.

At present, the regulation strategy for the uniform distribution of zinc ions has been widely reported. And the regulation strategies including artificial protective layer,^[^ ^7^ ^]^ alloy anode,^[^ ^8^ ^]^ 3D zinc anode,^[^ ^9^ ^]^ and electrolyte additives^[^ ^10^ ^]^ have been proven to be effective in optimizing the zinc deposition behavior at the anode interface.^[^ ^11^ ^]^ 3D zinc anode is a direct and effective strategy to reduce zinc dendrites by providing more sufficient active sites to meet the deposition/stripping of zinc ions. Zinc foam, copper mesh‐supported zinc anode, carbon/zinc composite anode and metal‐organic framework/zinc composite anode are typical 3D zinc anodes.^[^ ^12^ ^]^ The porous structure with high surface area is beneficial to the uniform electric field distribution and the circulation of zinc ions, thereby achieving the purpose of reducing the local current density. In addition, an artificial protective layer is a simple and effective means of protection thanks to simple operation and controllable process parameters. The existence of the artificial protective layer forms an effective isolation effect between the anode interface and the weak acid electrolyte, which significantly alleviates the erosion of the electrode interface. Some artificial protective layers have ion selectivity, which can uniform the electric field distribution near the interface and selectively allow zinc ions to pass through, thereby achieving the effect of regulating the uniform flow of zinc ions to reduce the possibility of rapid growth of zinc dendrites at local high electric fields and the occurrence of side reactions.^[^ ^13^ ^]^

The in situ protective layer prepared at the zinc anode interface by a series of methods (electrochemical deposition, chemical vapor deposition, physical vapor deposition, chemical replacement, in situ chemical reaction, etc.) has a strong chemical structure and excellent stability.^[^ ^14^ ^]^ A large number of zinc‐based materials have been proven to have a significant effect on inhibiting zinc anode dendrites, such as ZnO, ZnF_2_, and Zn_3_(PO_4_)_2_.^[^ ^15^ ^]^ The thickness of the interface protective layer based on this process is highly controllable through the flexible adjustment of process parameters. This superiority is superior to the traditional manual coating. It is a promising solution for aqueous zinc ion batteries to eliminate dendrites and side reactions at a high rate and achieve large capacity. Zhang et al. reported a multifunctional ZnSe protective layer that can simultaneously prevent dendrites and hydrogen evolution.^[^ ^16^ ^]^ The low Zn affinity of ZnSe and the unbalanced charge distribution at the interface can promote the uniform distribution of Zn^2+^ and accelerate the migration and deposition of Zn^2+^ to achieve dendrite‐free behavior. In addition to remarkable rate performance and cycle stability, the Zn@ZnSe||Zn@ZnSe symmetrical battery still maintains low polarization operation at a high current density of 10 mA cm^−2^. It is worth noting that the construction of 3D zinc anode based on protective interface and electrode structure is also an effective strategy to inhibit zinc dendrites. Yang et al. designed an interface material composed of a forest‐like 3D zinc‐copper alloy with an engineering surface to suppress dendrite growth on the anode surface of aqueous zinc‐ion batteries.^[^ ^17^ ^]^ The results show that the 3D nanostructured surface of the zinc‐copper alloy is beneficial to effectively adjust the reaction kinetics of the zinc plating/stripping process, thereby achieving high‐performance persistent water‐based zinc ion batteries in aqueous electrolytes containing monocations and dications. Yu et al. prepared a lightweight flexible 3D carbon nanofiber structure with uniform zinc seed (CNF‐Zn) by using bacterial cellulose to construct a zinc anode with highly reversible zinc electroplating/stripping ability.^[^ ^9^ ^]^ The prepared 3D CNF‐Zn has a porous interconnection network that can significantly reduce the local current density, and the functional Zn seeds can provide uniform nuclei to guide uniform zinc deposition behavior. Benefiting from the synergistic effect of the zinc seed and the 3D porous framework in the flexible CNF‐Zn host, this 3D zinc anode maintains excellent coulombic efficiency over a long cycle.

**Figure 1:**
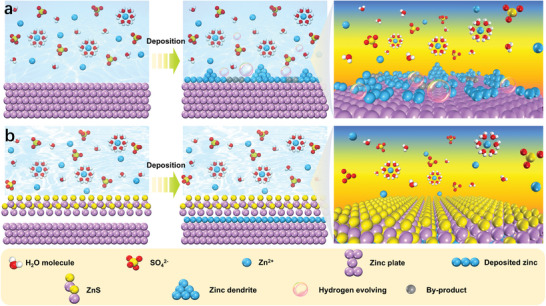
Schematic illustration of the deposition process of Zn^2+^ cations at the interface of a) Zn and b) ZnS@3D‐Zn anode.

In this work, we first created a large number of uniform 3D craters on the surface of the zinc anode by ultrasonic etching to provide more effective active sites for zinc deposition. After that, a zinc sulfide layer with optimized electron arrangement on the surface of the zinc anode was prepared in situ at the electrode interface using zinc foil as the active zinc source. The stable ZnS protective layer well regulates the interfacial electric field and the migration of Zn^2+^, thereby significantly promoting the homogenization of zinc ion flux to achieve Zn^2+^ dendrite‐free deposition (**Figure** **1**). It is worth noting that the ZnS@3D‐Zn||ZnS@3D‐Zn symmetric battery can maintain a long cycle of more than 3000 h at a current density of 8 mA cm^−2^, and achieve high coulombic efficiency of 99.6% in the Zn||Cu battery. In addition, the Zn||PC full cell assembled based on ZnS@3D‐Zn anode achieves better output performance in 3000 cycles. In summary, this research strategy opens up a new path for further enhancing battery performance and promoting the industrial application of aqueous zinc ion batteries.

## 2 Results and Discussion

### 2.1 Material Fabrication and Characterization

**Figure 2:**
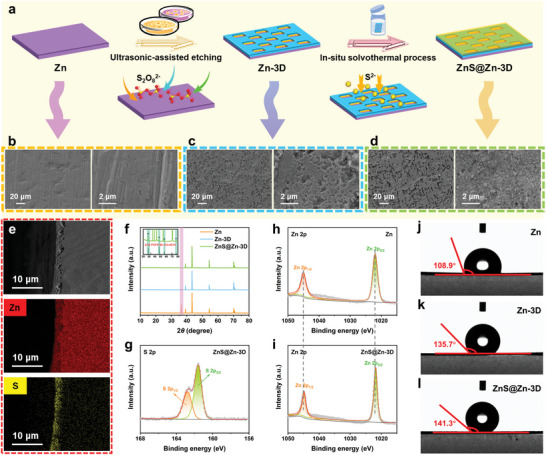
a) The schematic diagram of the preparation process of ZnS@Zn‐3D composite foil. Field‐emission scanning electron microscopy (FESEM) images of b) Zn, c)Zn‐3D, and d) ZnS@Zn‐3D foil. e) The cross‐section FESEM images of ZnS@Zn‐3D composite foil and the corresponding element distribution. f) X‐ray diffraction (XRD) patterns of Zn, Zn‐3D and ZnS@Zn‐3D foil. g) High‐resolution X‐ray photoelectron spectroscopy (XPS) spectra of S 2s of ZnS@Zn‐3D composite foil. High‐resolution XPS spectra of Zn 2p in h) Zn and i) ZnS@Zn‐3D composite foils. Contact angle test results of j) Zn, k) Zn‐3D, and l) ZnS@Zn‐3D foil.

The preparation process of the ZnS@3D‐Zn composite anode is shown in **Figure** **2**a. The surface of the commercial zinc foil is initially activated through a short‐term ultrasonic etching process. In this process, ammonium persulfate solution and zinc foil will undergo redox reaction. Ammonium persulfate (NH_4_)_2_S_2_O_8_ and zinc foil were used as oxidant and reducing agent, respectively. Persulfate S_2_O_8_^2−^ in ammonium persulfate will oxidize zinc metal to sulfate. The chemical equation of the reaction can be expressed as: *Zn*(*s*)  +  (*NH*_4_)_2_ *S*_2_ *O*_8_(*aq*)  →  *ZnSO*_4_(*aq*)  +  (*NH*_4_)_2_ *SO*_4_(*aq*). Therefore, a large number of loose and porous pits are generated on the surface of the flat zinc foil after a short period of ultrasonic etching (Figure 2b,c; Figure S1, Supporting Information), and many inert sites on the surface of the zinc foil are activated, which provides sufficient effective active sites for the deposition of zinc ions. The results of confocal laser scanning microscopy (CLSM) show that the etching gully cross‐section of Zn‐3D surface is U‐shaped with a depth of ≈2.8 µm. The front SEM images of the anode show that most of the gullies are in a long strip, and their length and width are mainly concentrated in 6–8 and 10–18 µm, respectively. After that, Zn‐3D was further treated by in situ hydrothermal strategy, zinc foil was used as zinc source to combine with S^2−^ and form ZnS on the surface.^[^ ^18^ ^]^ Although the surface of the zinc foil still presents a loose and porous concave structure, the high‐resolution FESEM image shows that the surface of ZnS@Zn‐3D is covered with a large number of ZnS nanoparticles (Figure 2d; Figure S2, Supporting Information). The cross‐section FESEM image of ZnS@Zn‐3D composite foil and the significant distribution of Zn and S elements further confirmed the synthesis of ZnS protective layer (Figure 2e). The entire protective layer is composed of stacked ZnS nanoparticles, and the ZnS protective layer is measured in detail. It can be seen from the measurement results that the diameter of ZnS nanoparticles on the surface of ZnS@Zn‐3D composite anode is mainly concentrated at 36.6–92.4 nm. The average diameter of these ZnS nanoparticles is 62.015 nm according to the Gaussian fitting results. In addition, the thickness of the protective layer composed of ZnS nanoparticles in the ZnS@Zn‐3D composite anode prepared based on this work is ≈2.1 µm (Figure S3, Supporting Information). This micron‐level protective layer can not only effectively prevent the high‐rate ion transport limitation caused by the excessive thickness of the protective layer, but also the sufficient gap structure between ZnS nanoparticles is very helpful for the efficient and orderly transport of zinc ions. The XRD patterns show that the (002) of Zn‐3D and ZnS@Zn‐3D has a significant decrease (Figure 2f; Figure S4, Supporting Information), indicating that the Zn (002) crystal plane is preferentially consumed during the entire reaction and exhibits a weak corrosion inhibition ability to ammonium persulfate. In addition to the obvious characteristic peak of Zn, the remaining characteristic peaks of ZnS@Zn‐3D are highly consistent with the standard card (PDF# 96‐153‐8618) of sphalerite‐type ZnS.^[^ ^19^ ^]^

The XPS spectra of Zn and ZnS@Zn‐3D composite foils are shown in Figure S5 (Supporting Information). The high‐resolution XPS spectra of S 2p show that ZnS@Zn‐3D has obvious peaks at 161.9 and 163.0 eV (Figure 2g), which belong to S 2p3/2 and S 2p1/2, respectively.^[^ ^20^ ^]^ The XPS peaks of Zn 2p spectrum of ZnS@Zn‐3D at 1021.8 and 1044.9 eV correspond to Zn 2p3/2 and Zn 2p1/2 (Figure 2h), respectively, which are slightly offset from the binding energy of Zn 2p in Zn at 1022.1 eV (Zn 2p3/2) and 1045.1 eV (Zn 2p1/2) (Figure 2i), which is mainly attributed to the stronger Zn‐S polar bond in ZnS.^[^ ^21^ ^]^ The contact angle test results show that the Zn foil has the smallest contact angle, and the contact angle of Zn‐3D and ZnS@Zn‐3D gradually increases (Figure 2j–l). A larger contact angle means that the material has better hydrophobicity. The contact angle of Zn‐3D is significantly larger than that of Zn, which should be due to the loose and porous pit structure on the surface. The further increased contact angle of ZnS@Zn‐3D is attributed to the ZnS nanostructures on the surface.

**Figure 3:**
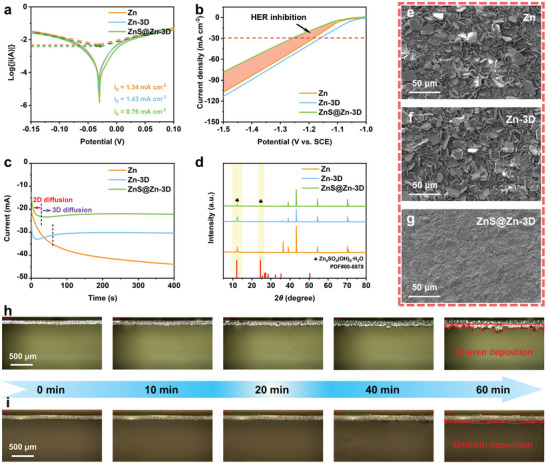
Electrochemical tests of Zn, Zn‐3D, and ZnS@Zn‐3D anodes: a) Linear polarization curves. b) Linear sweep voltammetry (LSV) curves at a scan rate of 5 mV s^−1^. c) Chronoamperometry profiles at an overpotential of −150 mV. d) XRD patterns and corresponding e–g) surface FESEM morphologies of Zn, Zn‐3D, and ZnS@Zn‐3D anodes immersed in 2 m ZnSO_4_ electrolyte for 10 days. In situ optical microscopy of h) Zn and i) ZnS@Zn‐3D anodes deposited in 2 m ZnSO_4_ electrolyte.

In order to further understand the effect of ultrasonic etching and ZnS nano‐protective layer on the surface of Zn anode, the linear polarization curves and linear sweep voltammetry curves of Zn, Zn‐3D, and ZnS@Zn‐3D anodes were first tested. The ZnS@Zn‐3D anode not only exhibits a more positive direction of corrosion voltage (**Figure** **3**a) but also its corrosion current density (0.76 mA cm^−2^) is significantly lower than that of Zn (1.34 mA cm^−2^) and Zn‐3D anode (1.43 mA cm^−2^). It is worth noting that the corrosion current density of Zn‐3D anode is slightly higher than that of Zn, which is attributed to more directly exposed zinc active sites. The results show that the ZnS nano‐protective layer can act as a corrosion inhibitor and reduce the side reaction of the zinc anode. Figure 3b is a linear sweep voltammetry curve of hydrogen evolution reaction on Zn, Zn‐3D, and ZnS@Zn‐3D anodes. In the voltage range of −1.5 to ‐1 V, the current density of the hydrogen evolution reaction of the ZnS@Zn‐3D anode is significantly reduced, and the voltage hysteresis effect is obvious at the same hydrogen evolution current density, indicating that the ZnS nano‐protective layer inhibits the hydrogen evolution reaction of the zinc anode.^[^ ^22^ ^]^ In order to elucidate the mechanism of zinc deposition behavior, chronoamperometry measurements were performed on symmetrical cells based on Zn, Zn‐3D, and ZnS@Zn‐3D anodes (Figure 3c). When the overpotential is −150 mV, the current of the pure zinc symmetrical battery increases by more than 400 s, and the current growth rate gradually slows down, indicating that the 2D plane diffusion of Zn^2+^ on the surface of Zn is slow over time.^[^ ^23^ ^]^ The Zn‐3D and ZnS@Zn‐3D symmetric cells exhibit a stable 3D diffusion process after 28 s and 60 s 2D planar diffusion nucleation, respectively. The results show that the ultrasonic etching treatment helps to inhibit the 2D diffusion of Zn^2+^ and promote the uniform nucleation of Zn^2+^. The ZnS nano‐protective layer further enhances this beneficial effect, which is very beneficial to inhibit the formation of Zn dendrites.

In order to study the inhibition effect of ZnS nano‐protective layer on the anodic side reaction in ZnSO4 electrolyte, the XRD spectra and SEM images of Zn, Zn‐3D, and ZnS@Zn‐3D immersed in electrolyte for 10 days were characterized. As shown in Figure 3d, XRD results show that the surface of Zn, Zn‐3D, and ZnS@Zn‐3D has different degrees of damage after immersion, among which the anode of Zn and Zn‐3D is particularly serious, while the surface of ZnS@Zn‐3D has only slight damage. According to the comparison of standard cards, the by‐product was identified as ZnSO_4_·3Zn(OH)_2_·H_2_O.^[^ ^24^ ^]^ This conclusion was further confirmed based on FESEM images that a large number of flake by‐products appeared on the surface of Zn and Zn‐3D anodes immersed in the electrolyte (Figure 3e–g; Figure S6, Supporting Information), showing obvious signs of corrosion and passivation. These results indicate that the ZnS nano‐protective layer effectively reduces the generation of by‐products and thus inhibits the corrosion of zinc foil.

### 2.2 Mechanistic Study of Zn Plating Kinetics and Zn^2+^ Migration

**Figure 4:**
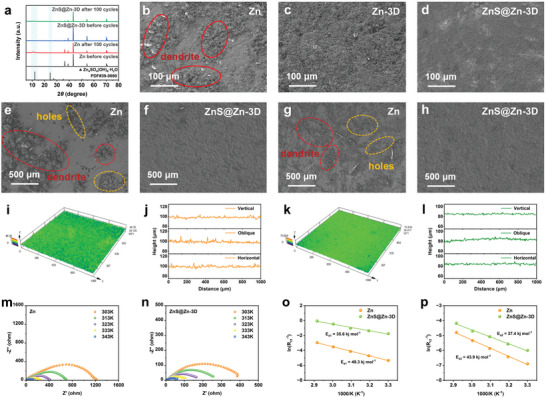
a) XRD spectra of Zn and ZnS@Zn‐3D anodes after 100 cycles at 1 mA cm^−2^ and 1 mAh cm^−2^. SEM images of b) Zn, c) Zn‐3D, and d) ZnS@Zn‐3D anodes after 100 cycles at 1 mA cm^−2^ and 1 mAh cm^−2^. SEM images of e) Zn and f) ZnS@Zn‐3D after 20 cycles at 0.25 mA cm^−2^ and 1 mAh cm^−2^. SEM images of g) Zn and h) ZnS@Zn‐3D after 20 cycles at 4 mA cm^−2^ and 1 mAh cm^−2^. The 3D images of CLSM and the corresponding line roughness of i and j) Zn and k and l) ZnS@Zn‐3D anodes after 100 cycles. The Nyquist spectra of m) Zn and n) ZnS@Zn‐3D symmetrical cells at different temperatures of 30 – 70 °C before cycling. The comparison of activation energy (Ea) of o) Zn and(p) ZnS@Zn‐3D electrodes based on Arrhenius curve.

The optimized galvanizing ability of ZnS@Zn‐3D was investigated in a transparent Zn||Zn battery at 5 mA cm^−2^ by in situ optical microscopy (Figure 3h,i). Obvious zinc dendrites appear on the surface of pure zinc after electrodeposition in 2 m ZnSO_4_ aqueous solution for 60 min, which may lead to unstable energy storage and permanent short circuit of the battery. The ZnS@Zn‐3D surface exhibits a relatively smooth and dendrite‐free appearance, confirming its excellent dendrite suppression ability. The results of XRD in **Figure** **4**a further prove that the existence of ZnS nano‐protective layer greatly inhibits the formation of by‐products and provides a more stable interface. The morphologies of Zn, Zn‐3D, and ZnS@Zn‐3D after 100 cycles at 1 mA cm‐2 were analyzed based on an SEM test (Figure 4b–d; Figure S7, Supporting Information). The picture shows that there are many dendritic agglomerations and patches on the rough surface of the bare zinc electrode, which is extremely unfavorable for maintaining the long cycle of the battery. In contrast, the Zn‐3D surface has less dendritic agglomeration due to the abundant zinc nucleation sites. It is worth noting that ZnS@Zn‐3D has a smooth surface and maintains a high degree of integrity after 100 cycles without obvious by‐products or dendrites, which is due to the dual gain of ultrasonic etching of 3D interface and ZnS nano‐protective layer. The SEM images of Zn and ZnS@Zn‐3D anode after 0.25 and 4 mA cm^−2^ cycles obtained similar results (Figure 4e–h; Figures S8, S9, Supporting Information). Zn showed obvious pits and dendrites on the surface after cycling, while ZnS@Zn‐3D always maintained a flat and smooth surface.

The deposition process of ZnS@Zn‐3D was explored in detail at 2 mA cm^−2^. The deposition results showed that the peak intensity of the (002) crystal plane gradually increased after the electrode was silenced for 60 min (Figure S10a, Supporting Information), indicating that the (002) crystal plane is preferentially deposited in the initial zinc deposition stage. In addition, SEM images of different deposition time of ZnS@Zn‐3D electrode show that the gully zone on the electrode surface is preferentially filled during the whole deposition process (Figure S10b–d, Supporting Information). It is worth noting that the Zn‐3D electrode also shows a similar trend (Figure S11, Supporting Information), which can effectively avoid the aggregation of local dendrites on the surface, indicating that this surface 3D reconstruction strategy is beneficial to the growth of dendritic zinc dendrites. Unfortunately, the Zn electrode showed a disordered deposition state, and a large amount of zinc dendrite aggregates accumulated on the electrode surface after 60 min of zinc deposition (Figure S12, Supporting Information). The advantages of ZnS@Zn‐3D anode were further verified by the 3D images of laser confocal microscopy (CLSM) and the corresponding line roughness test results of Zn and ZnS@Zn‐3D electrodes after 100 cycles (Figure 4i–l). The ZnS@Zn‐3D anode has a flatter deposition surface and a smaller cross‐sectional roughness, corresponding to a more ordered and stable zinc deposition behavior.

The electroplating/stripping behavior of ZnS@Zn‐3D anode was studied by Nyquist curve (Figure S13, Supporting Information). The main resistance of charge transfer migration is the desolvation process of Zn^2+^.^[^ ^25^ ^]^ The measured activation energy (Ea) of the electrode was evaluated by the Nyquist plots at different temperatures of 30–70 °C before the calibration cycle (Figure 4m,n).^[^ ^26^ ^]^ According to the Arrhenius equation, the decrease in the Ea value of the ZnS@Zn‐3D electrode corresponds to the accelerated desolvation of hydrated zinc ions, which is beneficial to the improvement of ion transfer kinetics.^[^ ^27^ ^]^ The ZnS@Zn‐3D electrode exhibits lower Ea values in the low‐frequency and intermediate‐frequency regions (Figure 4o,p), indicating that the electrode has better electrochemical kinetic characteristics. In summary, the excellent charge transfer kinetics of the ZnS@Zn‐3D anode are not affected despite the presence of the ZnS nanointerface phase.

**Figure 5:**
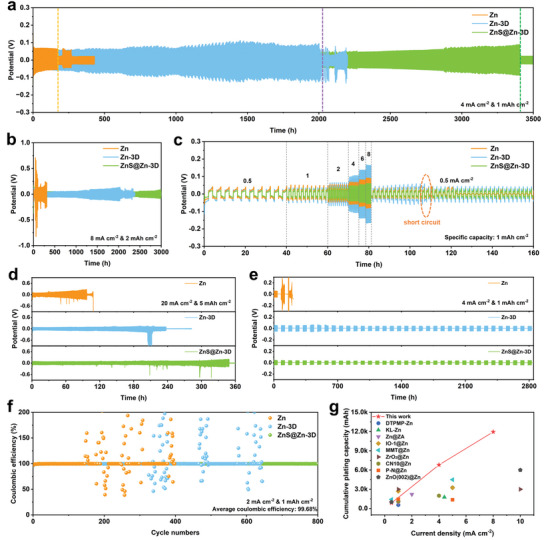
a) Galvanostatic charge/discharge cycling voltage profiles of Zn, Zn‐3D, and ZnS@Zn‐3D symmetrical cells at 4 mA cm^−2^ and 1 mAh cm^−2^. b) Galvanostatic charge/discharge cycling voltage profiles of Zn, Zn‐3D, and ZnS@Zn‐3D symmetrical cells at 8 mA cm^−2^ and 2 mAh cm^−2^. c) The rate performance of Zn, Zn‐3D, and ZnS@Zn‐3D symmetric cells at different current densities of 0.5–8 mA cm^−2^. d) Galvanostatic charge/discharge cycling voltage profiles of Zn, Zn‐3D, and ZnS@Zn‐3D symmetrical cells at 20 mA cm^−2^ and 5 mAh cm^−2^. e) Intermittent test of Zn, Zn‐3D, and ZnS@Zn‐3D symmetrical cells at 4 mA cm^−2^ and 1 mAh cm^−2^. f) The Coulombic efficiency stability of Zn||Cu asymmetric cells of Zn, Zn‐3D, and ZnS@Zn‐3D at 2 mA cm^−2^ and 1 mAh cm^−2^, respectively. g) Comparison of ZnS@Zn‐3D with previously reported work.^[^ ^25^, ^28^ ^]^

Thanks to the 3D reconstruction of the zinc surface and the ZnS nano‐protective layer, the ZnS@Zn‐3D electrode exhibits better electrochemical stability. As shown in **Figure** **5**a and Figures S14, S15 (Supporting Information), the optimized ZnS@Zn‐3D symmetrical cell exhibits excellent long‐period constant current cycling stability at 0.5 and 4 mA cm^−2^, and its life is as high as 3400 h. The voltage curve of Zn symmetrical cell at 4 mA cm^−2^ after 175 h can be observed with obvious fluctuation of battery failure. It is worth noting that the cycle life of Zn‐3D symmetric cell at 4 mA cm^−2^ is nearly 2000 h, which is significantly improved compared with Zn symmetric battery. Even at a high current density of 8 mA cm^−2^ (Figure 5b; Figure S16, Supporting Information), the ZnS@Zn‐3D symmetrical cell still maintains a service life of >3000 h. The ZnS@Zn‐3D symmetric cell exhibits the lowest nucleation voltage and nucleation overpotential at 0.5 mA cm^−2^ in the first cycle (Figure S17, Supporting Information), which is superior to Zn and Zn‐3D electrodes. Smaller nucleation voltage and lower nucleation voltage fluctuation correspond to a more stable reversible galvanizing process.^[^ ^29^ ^]^ It cannot be ignored that the ZnS@Zn‐3D symmetrical cell still has the best electrochemical zinc plating behavior at a high current density of 8 mA cm^−2^ (Figure S18, Supporting Information), which is conducive to the long‐term stable cycle of the electrode.

The rate performance of Zn, Zn‐3D, and ZnS@Zn‐3D symmetric cells in the current density range of 0.5–8 mA cm^−2^ were further evaluated. As shown in Figure 5c, when the current density increases from 0.5 to 10 mA cm^−2^, the ZnS@Zn‐3D symmetrical cell always maintains a low polarization voltage and a stable voltage distribution. When the current density is restored to 0.5 mA cm^−2^, the ZnS@Zn‐3D symmetric cell can obtain a normal and stable polarization voltage. In summary, the ZnS@Zn‐3D symmetric cell has high reversibility and fast electroplating/stripping kinetics. On the contrary, when the current density increases to 4 mA cm^−2^, the polarization voltage of the Zn symmetrical cell will suddenly decrease and fluctuate violently and then short circuit, which is mainly due to the rapid growth of dendrites at high current density.^[^ ^30^ ^]^ Although the Zn‐3D symmetrical battery cycled normally throughout the process, its polarization voltage also fluctuated sharply during the cycle. In addition, the Zn‐3D symmetric cell exhibits a significantly larger maximum voltage when the current density is increased to 4 mA cm^−2^. Even at an ultra‐high current density of 20 mA cm^−2^ and a high capacity density of 5 mAh cm^−2^ (Figure 5d), the improvement of the service life of the ZnS@Zn‐3D symmetrical cell is obvious. Intermittent tests are often used to evaluate the stability of symmetrical batteries.^[^ ^31^ ^]^ Zn‐3D and ZnS@Zn‐3D symmetrical cells have maintained excellent stability during the test period beyond 2800 h at 4 and 1 mA cm^−2^ (Figure 5e). The construction of 3D interface and nano‐zinc sulfide modification layer is effective for maintaining electrode stability.

In a Zn||Cu asymmetric cell with a fixed plating capacity of 1 mAh cm^−2^, a current density of 2 mA cm^−2^, and a cut‐off voltage of 0.8 V, the reversibility of the plating/stripping behavior of zinc in different anodes was further studied. The ZnS@Zn‐3D||Cu asymmetric cell maintained a stable output state throughout the 800 cycle tests (Figure 5f). During the whole cycle, the average coulombic efficiency of ZnS@Zn‐3D||Cu asymmetric cell is as high as 99.68%, indicating efficient zinc utilization of ZnS@Zn‐3D anode. The Zn||Cu and Zn‐3D||Cu failed to complete the entire cycle test, revealing the excellent long‐term cycle stability and reversibility of the ZnS@Zn‐3D||Cu asymmetric cell. Figure S19 (Supporting Information) shows the voltage‐capacity curves of Zn||Cu, Zn‐3D||Cu, and ZnS@Zn‐3D||Cu asymmetric batteries, respectively. In the initial cycle stage, all asymmetric cells have relatively high nucleation voltage. The nucleation overpotential of ZnS@Zn‐3D||Cu at 2.0 mA cm^−2^ is 47 mV, it is worth noting that ZnS@Zn‐3D||Cu maintains a stable low potential state in the subsequent cycle. This nucleation overpotential is lower than that of Cu//Zn and Zn‐3D||Cu, indicating a lower Zn^2+^ deposition barrier.^[^ ^32^ ^]^ Similar results were obtained based on the first‐cycle cyclic voltammetry curve of the Zn||Cu asymmetric cells of Zn, Zn‐3D, and ZnS@Zn‐3D (Figure S20, Supporting Information), which further revealed the excellent electrochemical nucleation kinetics of the ZnS@Zn‐3D electrode. In short, the cumulative plating capacity of the ZnS@Zn‐3D electrode is better than many previous reports (Figure 5g).

### 2.3 Optimized Electrochemical Deposition Mechanism of Zn^2+^

**Figure 6:**
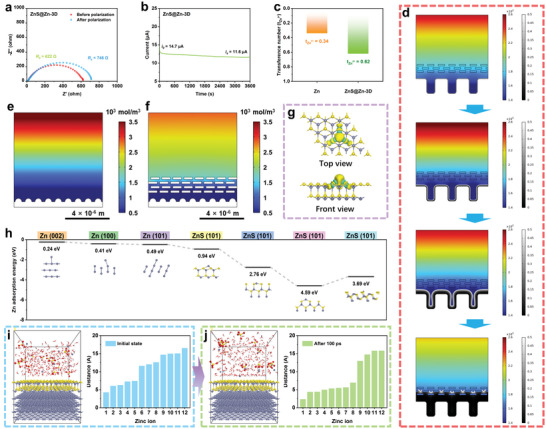
a) The Nyquist curve of ZnS@Zn‐3D symmetric cells before and after polarization, b) chronoamperometry profiles at an overpotential of 10 mV, c) calculated Zn^2+^ transfer numbers, and d) Zinc deposition diagram of Zn‐3D anode based on COMSOL finite element simulation. e,f) The concentration distribution of Zn^2+^ ions on the surface of Zn and ZnS@Zn‐3D anodes based on COMSOL finite element simulation. g) The electron density contribution diagram of Zn^2+^ at the interface of ZnS (yellow represents the acquisition of electrons, cyan represents the loss of electrons). h) The adsorption energies of Zn atoms on the crystal surfaces of Zn and ZnS. The molecular dynamics (MD) simulation of Zn^2+^ ions on the surface of ZnS@Zn‐3D anodes and the distance between Zn^2+^ ions and the surface: i) initial state, j) 100 ps.

In addition, the transfer number of Zn^2+^ (t_Zn_^2+^) of different anodes was calculated by the Bruce Vincent method to quantitatively estimate the transfer kinetics of Zn^2+^ on different anode surfaces.^[^ ^33^ ^]^ As shown in **Figures** **6**a–c and S21 (Supporting Information), the Zn^2+^ value increased significantly from 0.34 of Zn to 0.62 of ZnS@Zn‐3D, confirming the accelerated reaction kinetics. The improved Zn^2+^ transfer kinetics is helpful to optimize the deposition efficiency of Zn^2+^. The zinc deposition process of the 3D reconstructed zinc anode interface was verified by COMSOL finite element simulation. Thanks to the special concave gully structure, the Zn‐3D anode has a preferential deposition mechanism from the inside to the outside (Figure 6d; Figures S11,S22, Supporting Information). This not only provides a larger effective active area for the zinc deposition process but also helps to aggregate fewer planar zinc dendrites. The zinc concentration distribution on the surface of the zinc foil is further optimized by the modified ZnS nano‐protective layer. As shown in Figure 6e,f, an obvious concentration gradient of Zn^2+^ ions can be observed on the bare zinc anode. For conventional commercial pure zinc foil, the gradual accumulation of current density at the tip is limited by the “tip effect”, which is extremely unfavorable to the long‐term stable electroplating of the anode. The distribution of Zn^2+^ ions and the electrolyte current density at the anode interface of ZnS@Zn‐3D are significantly more uniform thanks to the addition of the nano‐ZnS interface phase, which ensures the uniform deposition behavior of zinc. In order to avoid the influence of the existing dendritic protrusions on the accurate experimental results, the Zn^2+^ ion distribution of flat pure zinc and ZnS@Zn‐3D anode was simulated under the same conditions (Figure S23, Supporting Information), and the test results were consistent with the preliminary results. In addition, the severe local current density at the protrusion of the zinc surface is effectively avoided due to the shielding effect of ZnS nanolayer (Figure S24,, Supporting Information), which can effectively inhibit the crazy growth of zinc dendrites at the protrusion of the zinc anode surface.

The calculation results based on DFT show that the combination of S atoms and Zn atoms in ZnS@Zn‐3D balances the charge distribution at the ZnS interface (Figure 6g), which is conducive to the orderly diffusion of Zn^2+^ at the anode interface. In addition, the DFT calculation results confirm that ZnS has a stronger adsorption effect on Zn atoms (Figure 6h; Figures S25,S26, Supporting Information), which contributes to the rapid deposition of zinc ions during zinc plating. It is worth noting that the (200) crystal plane of ZnS has a significantly enhanced adsorption effect on H_2_O molecules (Figures S27‐S29, Supporting Information), which can also accelerate the desolvation behavior of hydrated zinc ions. MD simulation based on 2 m ZnSO_4_ electrolyte was used to reveal the diffusion behavior of Zn^2+^ ions at the interface of Zn and ZnS@Zn‐3D anode (Figures S30,S31, Supporting Information). The results show that the Zn^2+^ ions on the Zn interface are always away from the interface during 100 ps, which is not beneficial to the electroplating process of zinc ions (Figure S32, Supporting Information). The Zn^2+^ ions near the ZnS@Zn‐3D interface are obviously close to the interface, which is powerful for the sustainable deposition kinetics of Zn^2+^ (Figure 6i,j). The diffusion behavior of Zn^2+^ near the anode interface is analyzed in detail (Figure S33, Supporting Information). The Zn^2+^ near the Zn interface is in a state of disordered Brownian motion, and even far away from the Zn interface during the diffusion process (Figure S34, Supporting Information). The Zn^2+^ near the ZnS@Zn‐3D interface is rapidly adsorbed to the interface, and the superiority of the electrochemical kinetics of the ZnS@Zn‐3D anode is further confirmed by the optimized migration path and diffusion distance.

### 2.4 Electrochemical Properties of the Aqueous Zinc‐iodine Full Cells

**Figure 7:**
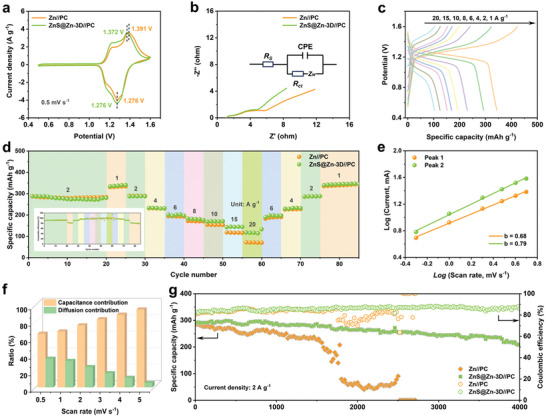
a) The CV curves of Zn//PC and ZnS@Zn‐3D//PC full cells at 0.5 mV s^−1^. b) The Nyquist curves of Zn//PC and ZnS@Zn‐3D//PC full cells (the equivalent circuit diagram is illustrated). c) The charge‐discharge curves of ZnS@Zn‐3D//PC full cells at different current densities. d) The rate performance of Zn//PC and ZnS@Zn‐3D//PC full cells (the illustration is the coulombic efficiency of the full cell). e) The relationship between log (peak current *i*) and log (scan rate *v*) (the relationship between current (*i*) and scan rate (*v*) can be determined by *i = av^b^* and *log (i) = log (a) + b log (v)* of ZnS@Zn‐3D//PC). f) The ratio of capacitance contribution and diffusion contribution at different scan rates. g) Long‐term cycle stability test of Zn//PC and ZnS@Zn‐3D//PC full cells at a current density of 2 A g^−1^.

The feasibility of the ZnS@Zn‐3D anode was evaluated by assembling full cells based on a biomass‐derived porous carbon cathode (Figures S35,S36, Supporting Information). In the voltage range of 0.5 – 1.6 V, the CV curves of Zn//PC and ZnS@Zn‐3D//PC full cells show overlapping profiles and similar redox peaks (**Figure** **7**a), indicating that the ZnS nano‐protective layer has little effect on the overall redox reaction of the battery. The obvious redox peak pair on the CV curve is attributed to the effect of I_3_^−^/I^−^ redox pair.^[^ ^34^ ^]^ It is worth noting that the redox peak potential of the ZnS@Zn‐3D//PC full cell was shifted to 1.276 and 1.372 V, respectively, compared with 1.276 and 1.391 V of the Zn//PC full cell. In addition, the ZnS@Zn‐3D//PC full cell has a higher exchange current density. The narrow redox potential range and high exchange current density are beneficial to the battery to obtain excellent redox reversibility and small polarization.^[^ ^35^ ^]^ In addition, the Nyquist diagram of the full cell shows that the ZnS@Zn‐3D//PC has a lower charge transfer resistance and a smaller low‐frequency slope than the Zn//PC full cell (Figure 7b; Figure S37, Supporting Information), which is conducive to better kinetic properties and rapid ion migration behavior. As shown in Figure 7c,d; Figure S38 (Supporting Information), the rate performance of the ZnS@Zn‐3D//PC full cell was evaluated. At low current density, Zn//PC and ZnS@Zn‐3D//PC full cells have similar performance, but ZnS@Zn‐3D//PC full cells have better rate performance at high current density. The ZnS@Zn‐3D//PC full cell has a reversible discharge specific capacity of 343.8 mAh g^−1^ at 1 A g^−1^ (based on the mass of the cathode active material). Even at an ultra‐high current density of 20 A g^−1^, the reversible discharge capacity of the ZnS@Zn‐3D//PC full cell still reaches 102.9 mAh g^−1^ and the Coulomb efficiency is close to 100%. It is worth noting that the electrode diffusion process has a high contribution to the capacity at low current density. In this process, the kinetics of the oxidation reaction and reduction reaction of iodine on the electrode surface may be slow, which leads to the incomplete conversion of iodine ions during charge and discharge, thus affecting the coulombic efficiency and the overall energy conversion efficiency of the battery. At the same time, this also explains that the battery in the Figure 7d illustration has a better Coulomb efficiency in high energy density.

From the fitting results of the peak current and scan rate in the CV curve of the battery, it can be seen that the energy storage process of the ZnS@Zn‐3D//PC full battery based on the biomass‐derived porous carbon cathode is dominated by the capacitance contribution and the diffusion contribution (Figure 7e,f; Figures S39,S40, Supporting Information). The detailed fitting results show that the capacitance contribution of the battery gradually dominates as the scanning rate increases, which helps the battery to provide excellent electrochemical kinetics at high rates and is also the main reason for the battery to maintain excellent rate performance at high current densities.^[^ ^36^ ^]^ In addition, the ZnS@Zn‐3D//PC full cell with modified anode can operate stably up to 4000 times at 2 A g^−1^. As a comparison, the Zn//PC full battery showed a significant capacity decay and subsequent short circuit at 2 A g^−1^ in the 1561th cycle (Figure 7g). Even at a high current density of 10 A g^−1^, the ZnS@Zn‐3D//PC full cell can operate stably for more than 10 000 cycles without significant capacity decay (Figure S41, Supporting Information). Therefore, these results prove that the improvement of the anode performance of zinc metal battery by ZnS@Zn‐3D composite anode is obvious.

## 3 Conclusion

In summary, the novel zinc anode based on 3D reconstruction and zinc sulfide nanolayer modification achieved excellent cycle stability and excellent service life. The 3D structure obtained based on the ultrasonic‐assisted etching strategy provides a large number of effective active sites for the zinc deposition process. The in situ ZnS interface phase further regulates the interfacial electric field and the efficient migration of Zn^2+^, thereby significantly promoting the homogenization of zinc ion flux. This dual‐gain protection can not only inhibit side reactions and delay the corrosion rate but also reduce the voltage hysteresis and nucleation overpotential, thereby achieving orderly diffusion and uniform deposition of Zn^2+^. The ZnS@3D‐Zn symmetric cell can maintain a long cycle of more than 3000 h at a current density of 8 mA cm^−2^. In addition, the full cell assembled based on ZnS@3D‐Zn anode achieved better output performance in the long‐term cycle. In summary, this work sheds light on the importance of reasonable interfacial modification for the development of dendrite‐free and stable zinc anode chemistry, which opens up a new path for promoting the development of zinc‐based batteries.

## Conflict of Interest

The authors declare no conflict of interest.

## Author Contributions

D.F.G. and B.Z. conceived the project and designed the experiments; D.F.G. and F.Y.L. contributed to sample preparation and experiments; D.F.G. carried out theoretical calculation and data collation, and completed the first draft writing and form analysis; B.Z. participated in the manuscript examination. All authors discussed the results and commented on the manuscript.

## Supporting information



Supporting Information

## Data Availability

The data that support the findings of this study are available from the corresponding author upon reasonable request.
