# Supplementary material for: Nano‐Zinc Sulfide Modified 3D Reconstructed Zinc Anode with Induced Deposition Effect Assists Long‐Cycle Stable Aqueous Zinc Ion Battery
Source: Adv Sci (Weinh). 2025 Jan 21;12(10):2417323. doi: 10.1002/advs.202417323 (PMC11904976; doi:10.1002/advs.202417323)
Supplement: Supplementary file 1 — Supporting Information [file ADVS-12-2417323-s001.docx]

**Supporting material**

Nano-Zinc Sulfide Modified Three-Dimensional Reconstructed Zinc Anode with Induced Deposition Effect Assists Long-Cycle Stable Aqueous Zinc Ion Battery

*Dongfang Guo**^1,2^, Fengyu Li^1,2^, Bin Zhang^1,2,*^*

^1^ School of Physics and Microelectronics, Zhengzhou University, Zhengzhou, 450001, China

^2^ School of Physics and Laboratory of Zhongyuan Light, Zhengzhou University, Zhengzhou, 450001, China

E-mail: [1184669315@qq.com](mailto:1184669315@qq.com) (D.F. G.); [fengyuli_zzu@163.com](mailto:fengyuli_zzu@163.com) (F.Y. L.); [zb1967@zzu.edu.cn](mailto:zb1967@zzu.edu.cn) (B. Z.)

**Experimental sections**

**Preparation of materials**

**Preparation of 3D-Zn foils.** The Zn foil with a thickness of 100 μm was washed with acetone and deionized water and dried at 60℃ for later use. After that, both sides of the zinc foil were polished with fine sandpaper (2000 mesh) to remove the zinc oxide layer, and then the polished zinc foil was immersed in a petri dish containing 0.5M (NH_4_)_2_S_2_O_8_ for ultrasonic treatment (40 kHz). It is worth noting that the back of the zinc foil is protected with kapton tape. After ultrasonic treatment, the zinc foil was thoroughly cleaned with deionized water, and the final zinc foil product was 3D-Zn.

**Preparation of ZnS@3D-Zn composite foils.** All reagents can be used directly without further purification. Firstly, 80 ml of mixed solution was configured with deionized water and polyethylene glycol at a volume ratio of 7:1. Then 1.78 g of K_2_S·5H_2_O powder was dissolved in the above mixed solution and stirred magnetically for 30 min. The final mixed solution and 3D-Zn foil (4 cm × 7 cm) were then transferred to a 100 mL Teflon-lined stainless-steel autoclave and kept at 120℃ for 20 h. The hydrothermally treated zinc foil was vacuum dried at 80℃ for 24 h to obtain the ZnS@3D-Zn composite foil. It is worth noting that the unetched side of the composite foil was sanded with fine sandpaper before use to avoid the influence of the protective layer on the test.

**Preparation of walnut shell-derived porous carbon (PC).** First, the walnut shell was cleaned with acetone and water ionized water in turn, and dried at 80℃ for later use. The walnut shell was carbonized in N_2_ atmosphere at 500℃ for 2 h, and then the walnut shell-derived carbon was mixed with KOH at a mass ratio of 1:3 and activated at 700℃ for 2 h in N_2_ atmosphere. After that, the residual KOH in the sample was washed with 3M HCl and washed with deionized water to neutral. Finally, PC cathode was obtained by vacuum drying the above powder at 60℃ for 12 h.

**Electrochemical Measurement**

**Symmetric cells and asymmetrical cells.** The symmetrical cell was assembled in an air environment in the form of CR2032-type coin cells. Glass fiber filter (GF-D, Whatman), zinc metal foil and 2M ZnSO_4_ aqueous solution as separator, electrode and electrolyte, respectively. In asymmetric cells, zinc foil and copper foil are used as working electrode and counter electrode respectively.

**Full Cell.** The full cell was assembled directly in air as a CR2032 coin cell. Glass fiber diaphragm (GF-D, Whatman), zinc foil and an aqueous solution of 2M ZnSO_4_ + 0.2M ZnI_2_ were used as the diaphragm, anode and electrolyte, respectively. A slurry consisting of WPC, carbon black and polyvinylidene fluoride (PVDF) mixed in N-methyl-2-pyrrolidone (NMP) solvent at a weight ratio of 8:1:1 was uniformly coated on a stainless-steel mesh and dried at 60℃ for 12 h as the cathode of the full cell. The dried electrode plates were cut into 12 mm diameter discs for spare parts, and the loading mass of a single disc was 2 mg cm^-2^.

**Electrochemical Characterization.** Galvanostatic charge/discharge curves, rate performance, and long-term cycling tests of the full cells at different current densities at room temperature were recorded on a LANHE CT3001A battery tester (Wuhan, China). The Electrochemical impedance spectroscopy (EIS) spectra (from 100 kHz to 0.01 Hz), Cyclic voltammetry (CV), Linear polarization curves and chronoamperometry (CA) were tested on CHI760E electrochemical workstation (Shanghai, China). The three-electrode system consists of a bare Zn or surface-modified Zn foil as the working electrode, a platinum foil as the counter electrode, and an Ag/AgCl electrode as the reference electrode. The CA tests were carried out in the three-electrode system at an overpotential of -150 mV. LSV was tested in the voltage range from -1.6 to -1 V at a scan rate of 5 mV s^-1^.

The EIS of symmetrical cells at different temperatures was measured. The linear relationship between charge transfer resistance (*R_ct_*) and temperature can be obtained by fitting the Arrhenius equation ^[1]^:

$\frac{1}{R_{ct}}=Aexp(\frac{-E_{a}}{RT})$ *(1)*

Where *R_ct_* is the charge transfer resistance, and *A*, *T*, *R* and *E_a_* are constants, Kelvin temperature, ideal gas constant and activation energy, respectively.

The Zn^2+^ transference number (t_Zn_^2+^) is calculated according to the Bruce Vincent method ^[2]^:

$t_{{Zn}^{2+}}=\frac{I_{s}\left( V-I_{0}R_{0} \right)}{I_{0}\left( V-I_{s}R_{s} \right)}$ *(2)*

The V is the applied potential of 10 mV, *I_0_* and *I_s_* are the initial current and steady-state current after CA measurements, and the *R_0_* and *R_s_* are the initial interface resistance and steady-state resistance after CA measurements.

**Characterizations**

Powder X-ray diffraction (XRD) patterns were conducted by the PANalytical Empyrean using Cu Kα-radiation (λ= 1.5418). The micromorphology of the samples was measured by field-emission scanning electron microscopy (FESEM, Thermo Scientific/Helios G4 CX) and high-resolution transmission electron microscopy (HRTEM, JEM-1400Flash). The surface element distribution was explored by energy dispersive X-ray spectroscopy (EDX). The surface chemical compositions were analyzed by X-ray photoelectron spectroscopy (XPS, AXIS SUPRA). Electrochemical testing was performed on an electrochemical workstation CHI760E (Shanghai, China) and LANHE CT3001A (Wuhan, China).

**Density functional theory (DFT) calculations**

The whole DFT calculation process is carried out by Material Studio 2020, using the DFT Semi-core Pseudopots with Dmol3 module. The Perdew-Burke-Ernzerhof (PBE) functional within the generalized gradient approximation (GGA) were applied to describe ionic cores and exchange-correlation effects, respectively. The vacuum gap between periodic images was set to 15 Å to avoid interaction. The convergence criteria for residual force and energy were set to 0.05 eV Å^-1^ and 1 × 10^-6^ eV, respectively.

The surfaces were built to investigate the adsorption energies of Zn. The adsorption energies of the Zn ion on the Zn and ZnS surfaces were calculated as follows:

$E_{ad}=E_{t}-E_{s}-E_{Zn}$ (3)

where E_t_, E_s_, E_Zn_ were the total energy of Zn adsorbed on the surface, the surface energy, and the energy of individual Zn atom, respectively. The charge density changes of the Zn adsorbed on the surface were calculated following the equation:

$\rho=\rho_{t}-\rho_{s}-\rho_{Zn}$ (4)

where ρ_t_, ρ_s_, ρ_Zn_ were the charge densities of Zn adsorbed surfaces, individual the surfaces and Zn atom, respectively. When the above convergence criteria were satisfied, the structure optimization was completed.

The free energy profile for the HER was obtained based on the computational hydrogen electrode (CHE) model proposed by Nørskov and co-workers. In this model, the free energy of proton-electron pair is equal to that of 1/2 H_2_(g). The free energy change for each fundamental step was determined by:

$\Delta G=\Delta E+E_{ZPE}+T\Delta S$ (5)

where *∆E* is the difference of electronic energy directly obtained from DFT simulation. *E_ZPE_* is the variation of zero-point energy (*ZPE*), *∆S* is the entropy (*S*) change, *T* is the temperature (T = 298.15K). The *ZPE* and *S* were obtained by the vibrational frequencies. For the molecular in gas phase, H_2_ (g), the *ZPE* and *S* were taken from *NIST* database.

**Molecular dynamics (MD) simulation**

MD calculation for 2M ZnSO_4_ aqueous solution on Zn and ZnS@3D-Zn anode surface. Zn (002) and ZnS (111) are typical crystal planes. The solid-liquid interface calculations for ZnSO_4_ aqueous solution on Zn and ZnS@3D-Zn anode surface were conducted with universal force field using the Forcite module in MS 2020. The dimensional lengths of the rectangular Zn slab are 26.13 Å × 27.15 Å in plane, which contains 7 layers of Zn (002). The dimensional lengths of the rectangular ZnS@3D-Zn slab are 26.13 Å × 27.15 Å in plane, which contains 4 layers of Zn (002) and 4 layers of ZnS (111). The ZnSO_4_ aqueous solution contains 12 Zn^2+^, 12 SO_4_^2-^ and 324 H_2_O molecules in one rectangular box with length scales of 26.13 Å × 27.15 Å × 14 Å. After geometry optimization, the aqueous solution was placed on the Zn (002) and ZnS (111) plane. The surface charge situations of Zn (002) and ZnS (111) plane were 0 C m^-2^. All MD calculations were performed under the NVT ensemble (T=298.0 K) with a time step of 0.1 ps and a total simulation time of 100 ps, during which simulation trajectories were recorded every 100 steps. The running time was long enough for system energy and temperature reaching stable. The temperature is controlling by a Nose-Hoover thermostat. The Ewald scheme and atom-based cutoff method with a radius of 12.5 Å were applied to treat electrostatic and van der Waals (vdW) interactions, respectively. The solid-liquid interfaces snapshots were displayed.

**Multi-physics field simulation**

The current density distribution (also known as Zn^2+^ flux distribution) and Zn^2+^ ion concentration distribution of Zn and ZnS@3D-Zn samples were numerically simulated by using "Cubic current distribution" in COSMOL Multiphysics. In the simplified two-dimensional model, the interlaced nanosheet structure was used to represent the ZnS interface. The size of the entire two-dimensional model for the current density distribution analysis is set to 8 μm × 8 μm. The top of the geometry represents the cathode, the anode is at the bottom, and the vertical wall is assumed to be the insulation boundary. The distance between the top and bottom electrodes is 7 μm. The two-dimensional deposition diagram of the Zn-3D anode was simulated. The zinc deposit is elliptic. The electrode surface reaction is given by Butler-Volmer kinetic expression. The simulation parameters are as follows: the ion diffusion coefficient of ZnSO_4_ electrolyte is set to 2 × 10^-9^ m^2^ s^-1^, the initial concentration of Zn^2+^ is set to 2 mol L^-1^.


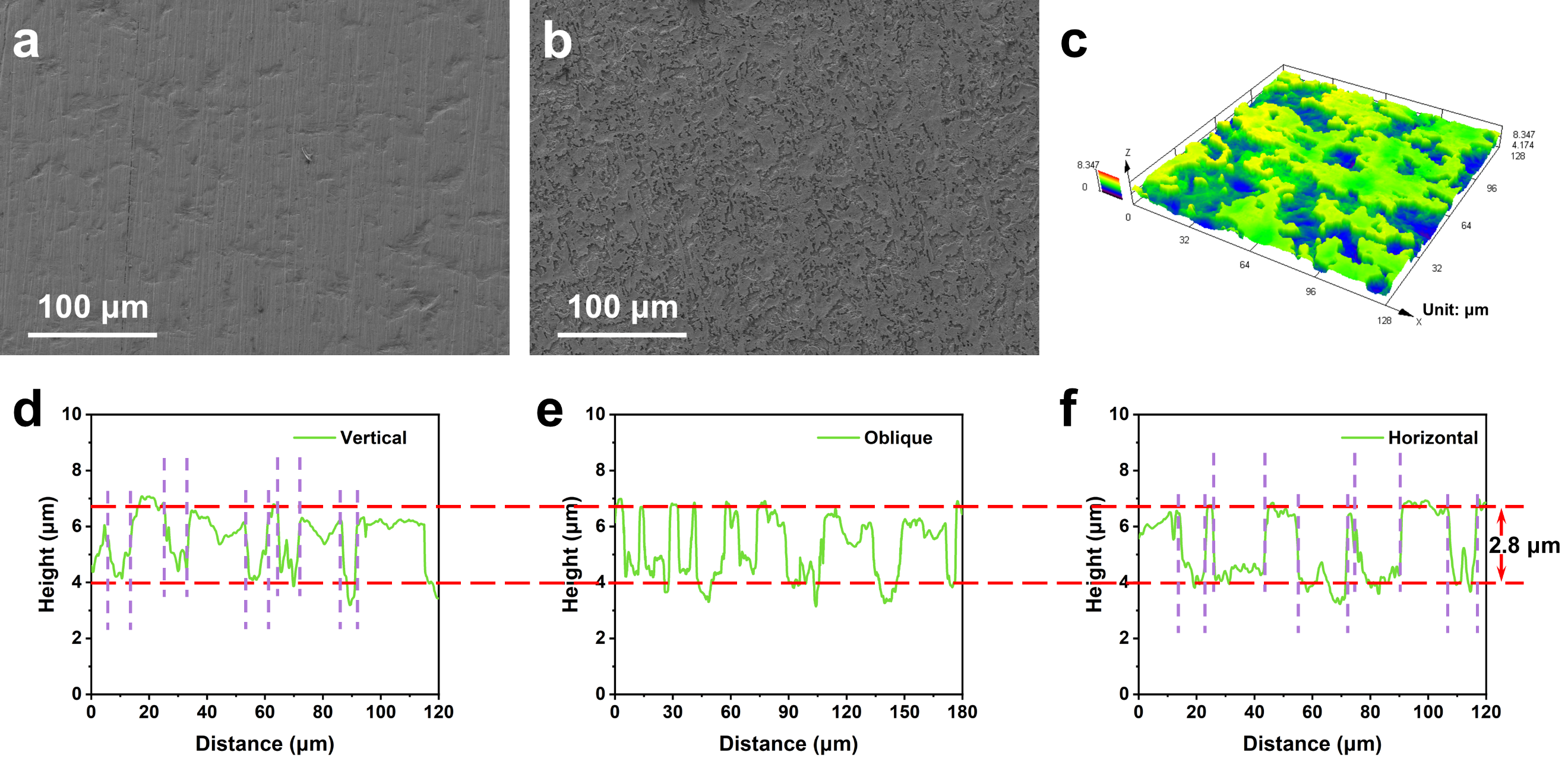


**Figure S1** FESEM images of (a) Zn and (b) Zn-3D surfaces. (c) The three-dimensional images of confocal laser microscopy (CLSM) and (d-f) the corresponding line roughness of Zn-3D anodes.


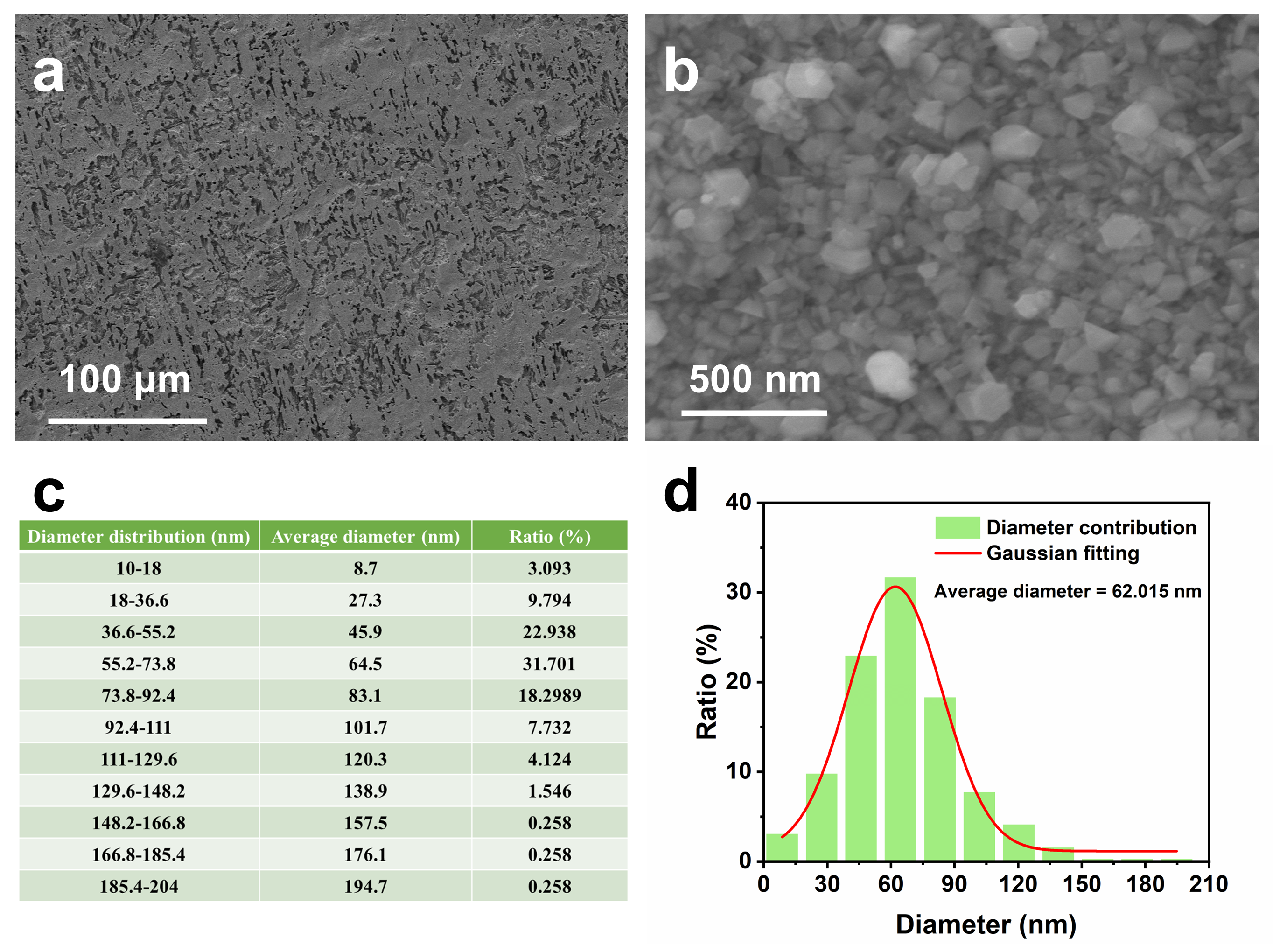


**Figure S2** (a, b) FESEM images of ZnS@Zn-3D composite foil surface. (c, d) The diameter statistics of ZnS nanoparticles on the surface of ZnS@Zn-3D anode and the corresponding Gaussian distribution.


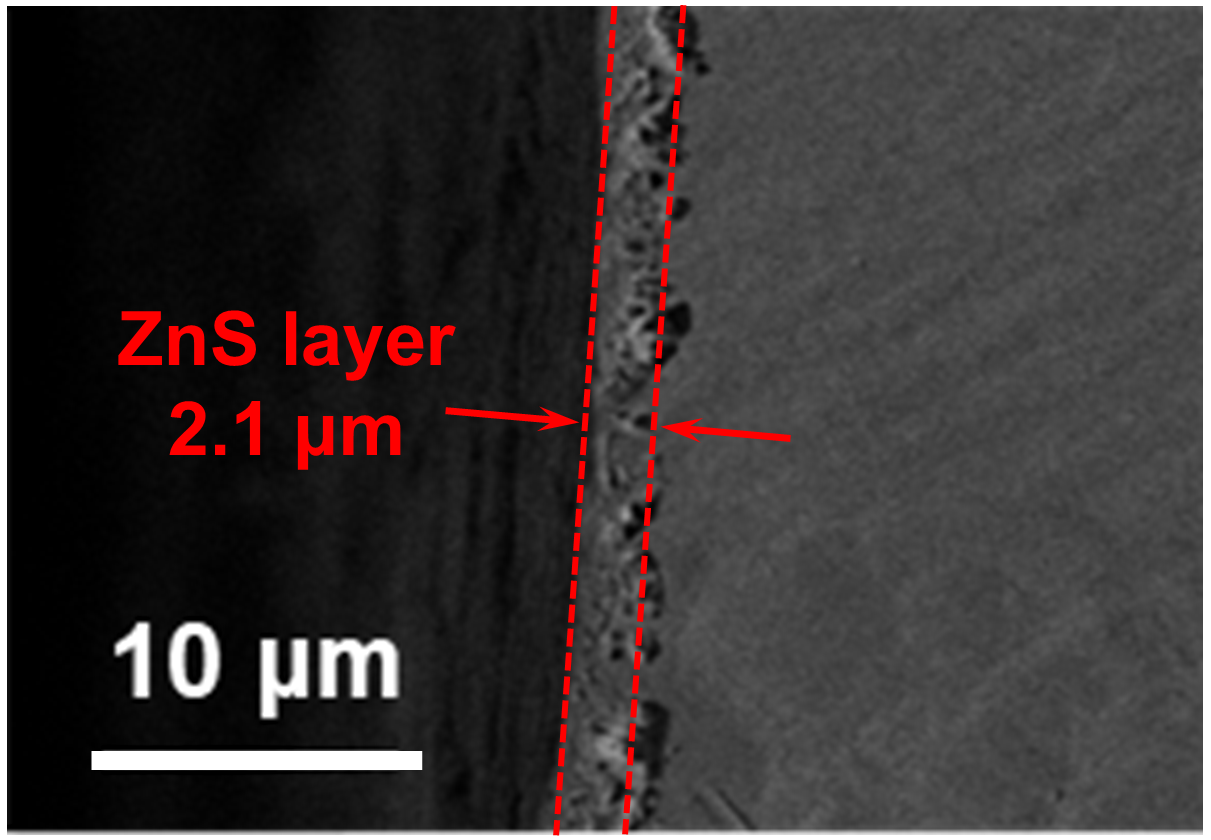


**Figure S3** FESEM images of ZnS@Zn-3D composite foils and the corresponding ZnS interface thickness.





**Figure S4** The comparison results of XRD pattern and ZnS standard card of ZnS@Zn-3D composite foils.





**Figure S5** XPS results of Zn and ZnS@Zn-3D composite foils.


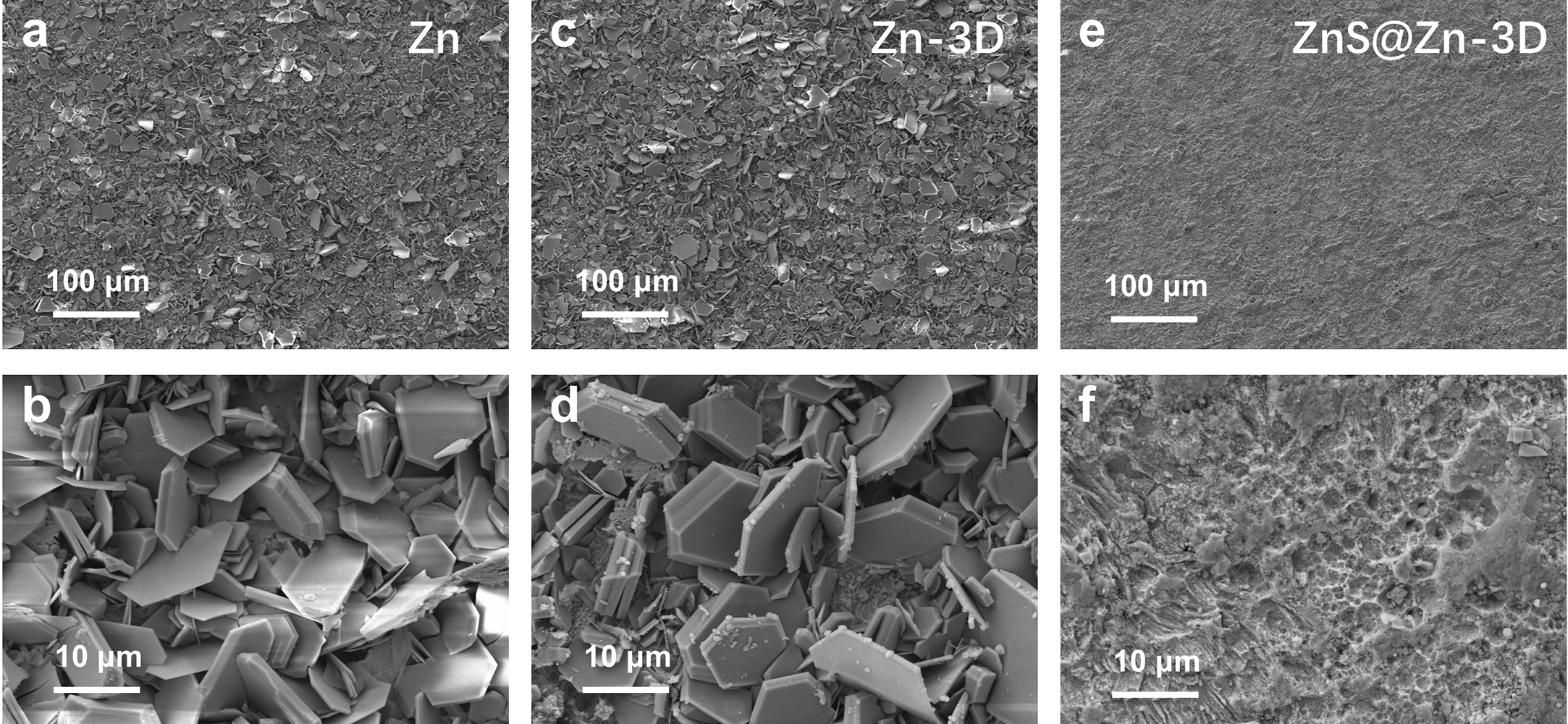


**Figure S6** The surface FESEM morphology of (a, b) Zn, (c, d) Zn-3D and (e, f) ZnS@Zn-3D electrodes immersed in 2M ZnSO_4_ electrolyte for 10 days.


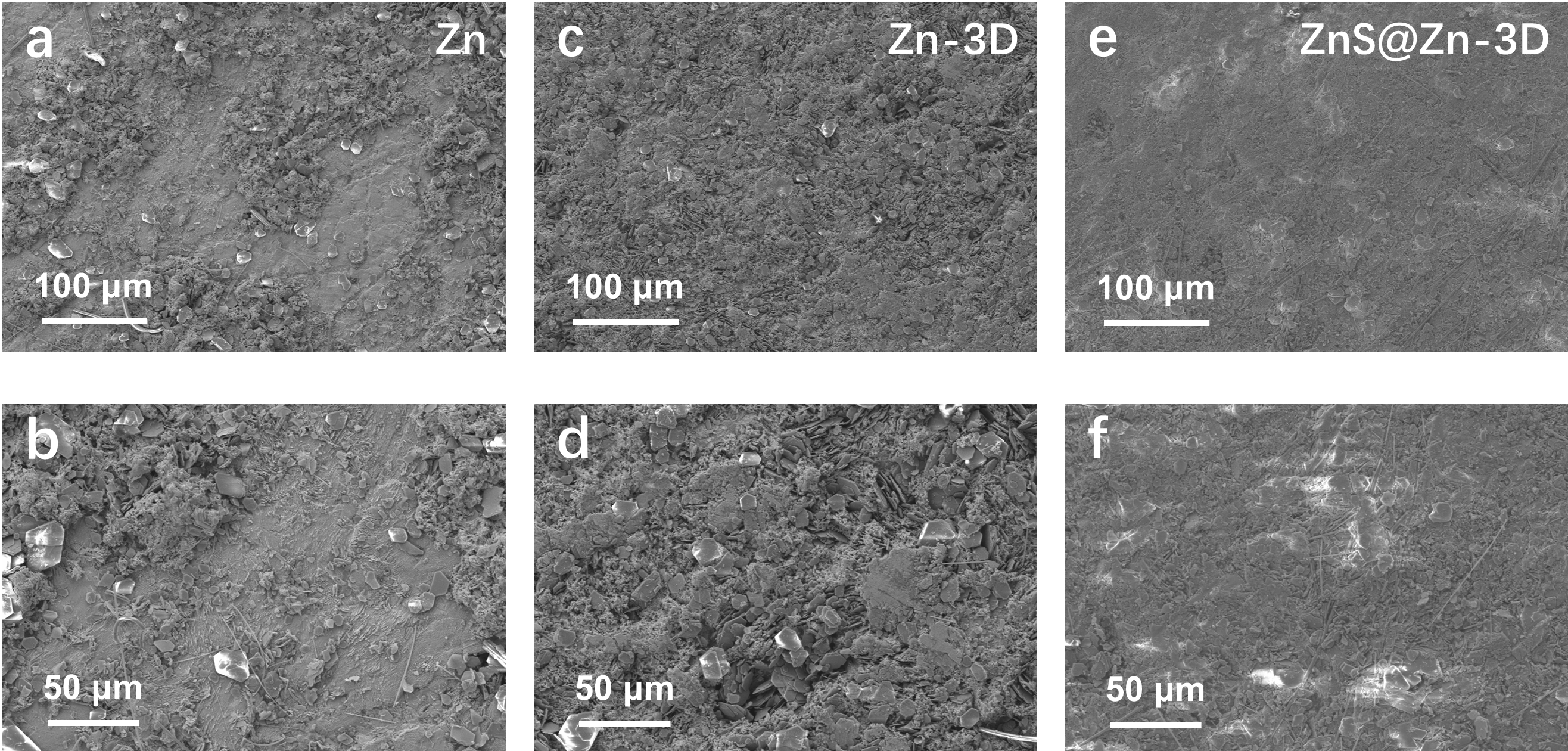


**Figure S7** SEM images of (a, b) Zn, (c, d) Zn-3D and (e, f) ZnS@Zn-3D anodes after 100 cycles at 1 mA cm^-2^ and 1 mAh cm^-2^.


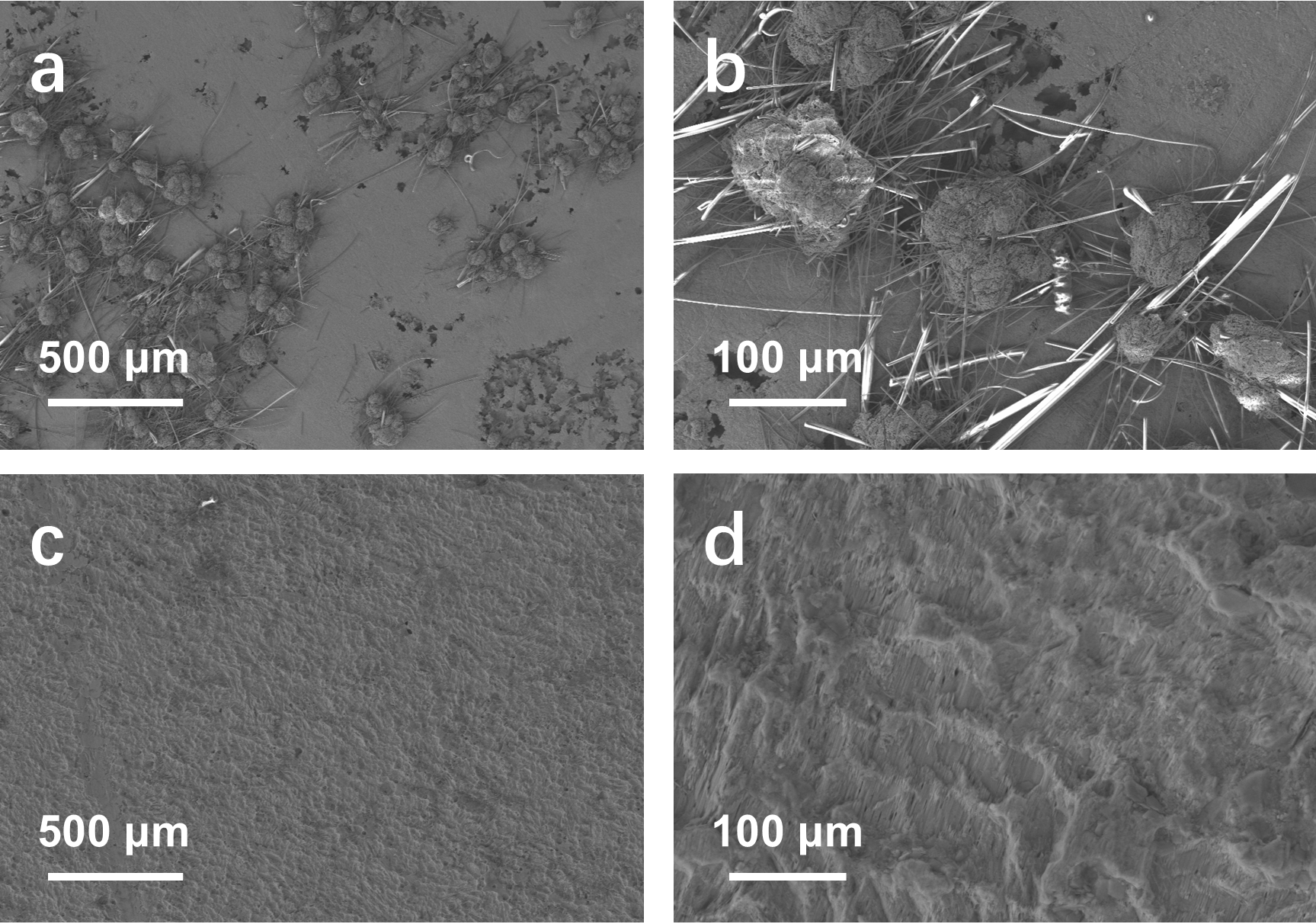


**Figure S8** SEM images of (a, b) Zn and (c, d) ZnS@Zn-3D anodes after 20 cycles at 0.25 mA cm^-2^ and 1 mAh cm^-2^.


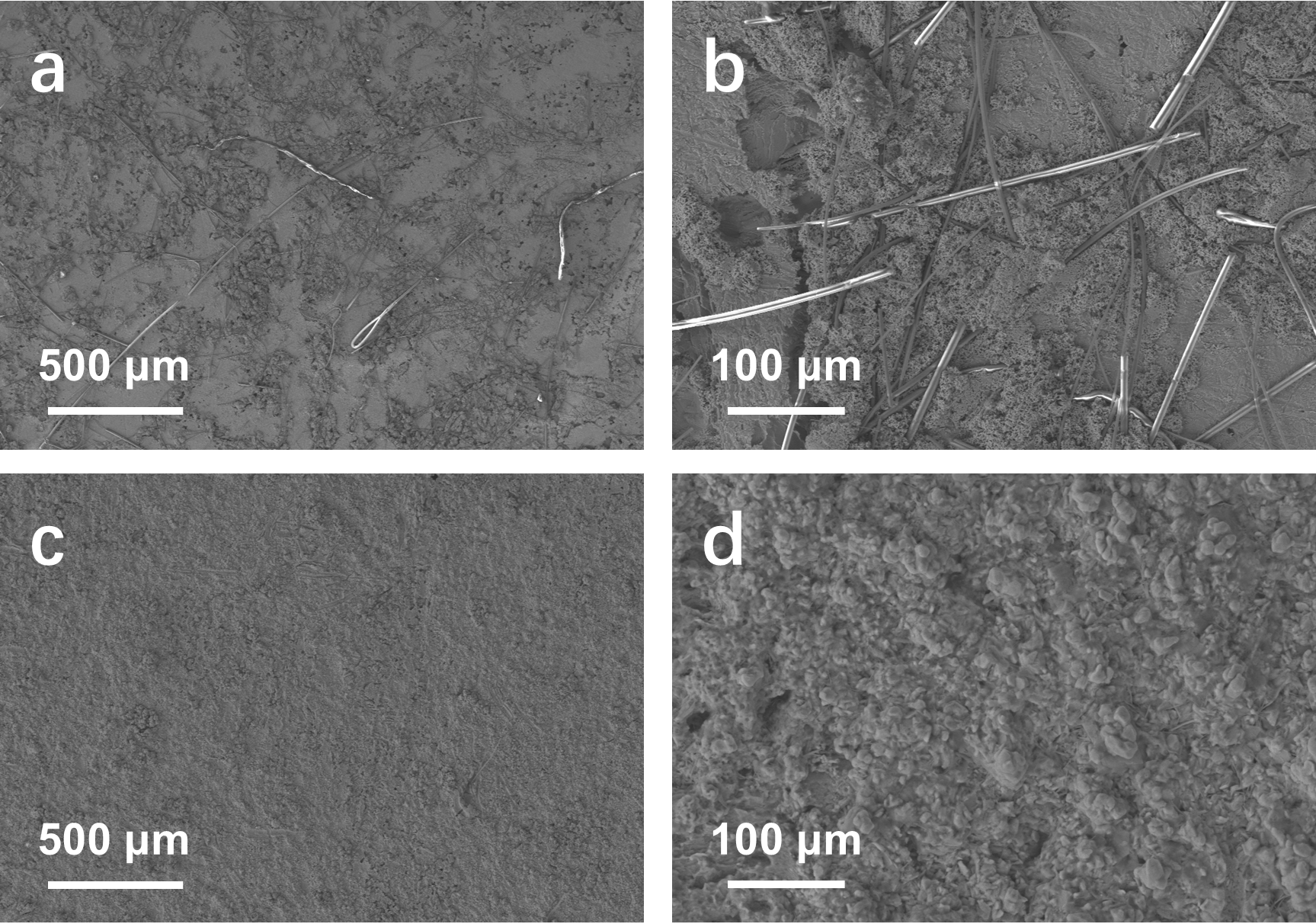


**Figure S9** SEM images of (a, b) Zn and (c, d) ZnS@Zn-3D anodes after 20 cycles at 4 mA cm^-2^ and 1 mAh cm^-2^.


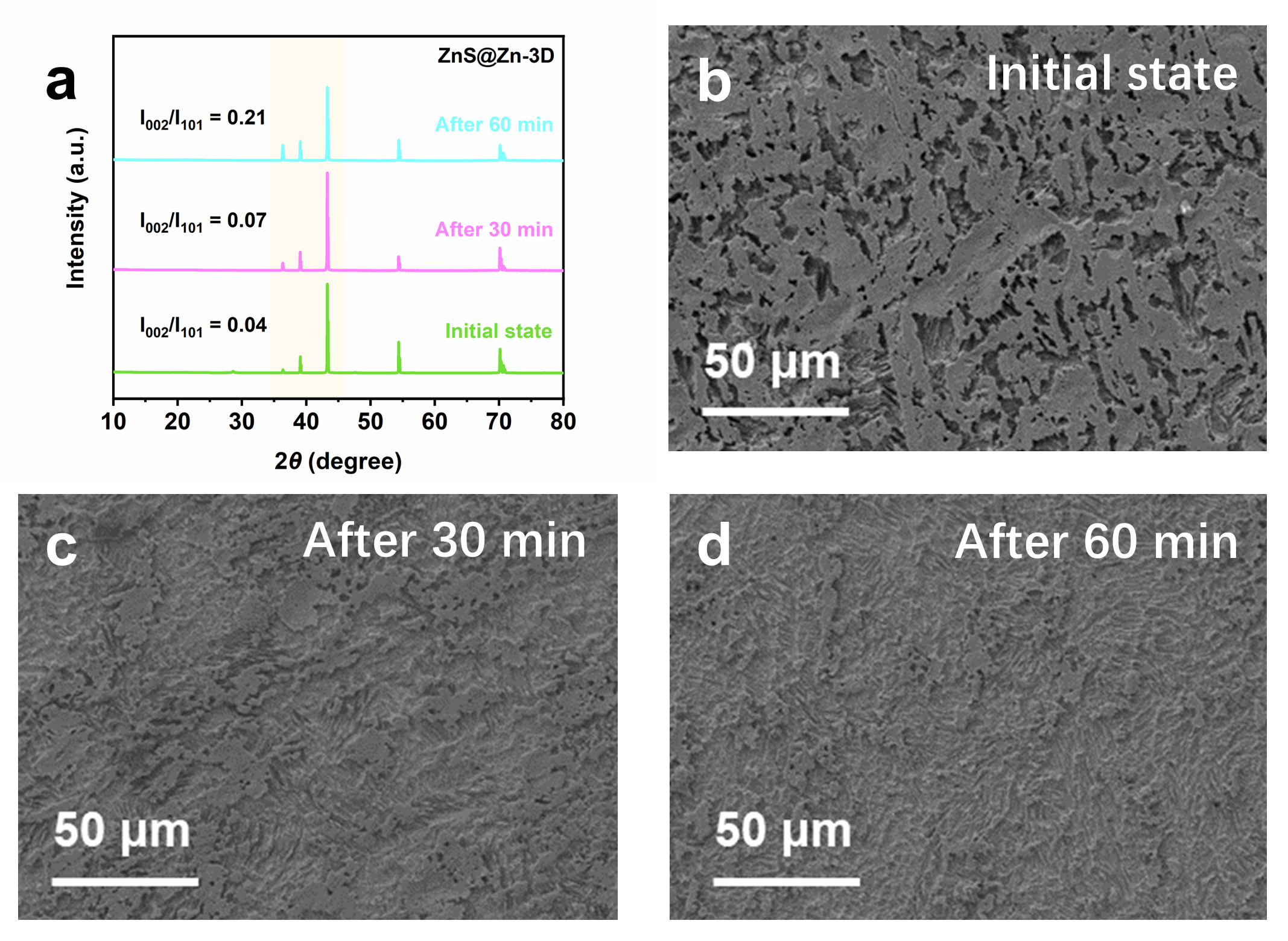


**Figure S10** The (a) XRD spectra and (b-d) SEM images of ZnS@Zn-3D anodes after cycling at 2 mA cm^-2^ for 0 min, 30 min and 60 min, respectively.


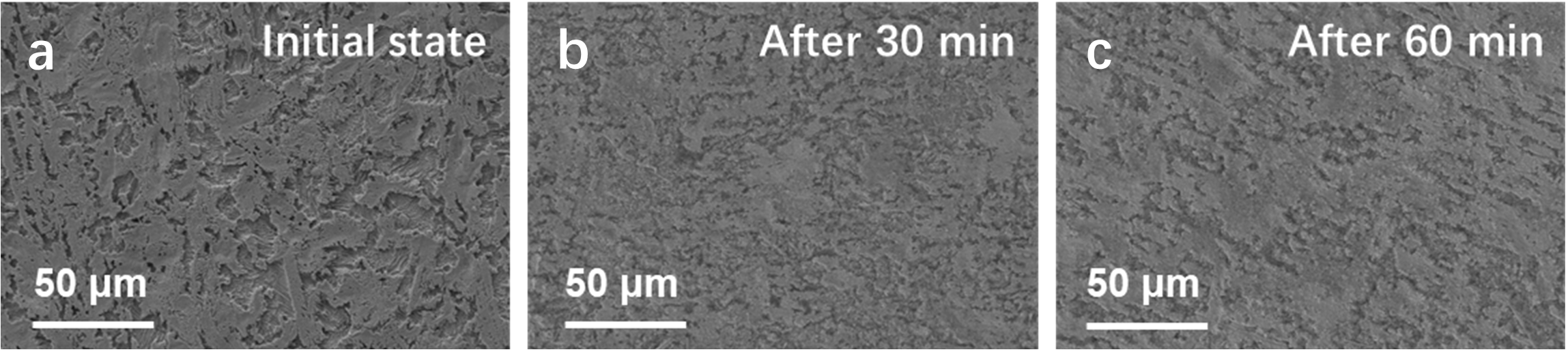


**Figure S11** (a-c) The SEM images of Zn-3D anodes after cycling at 2 mA cm^-2^ for 0 min, 30 min and 60 min, respectively.


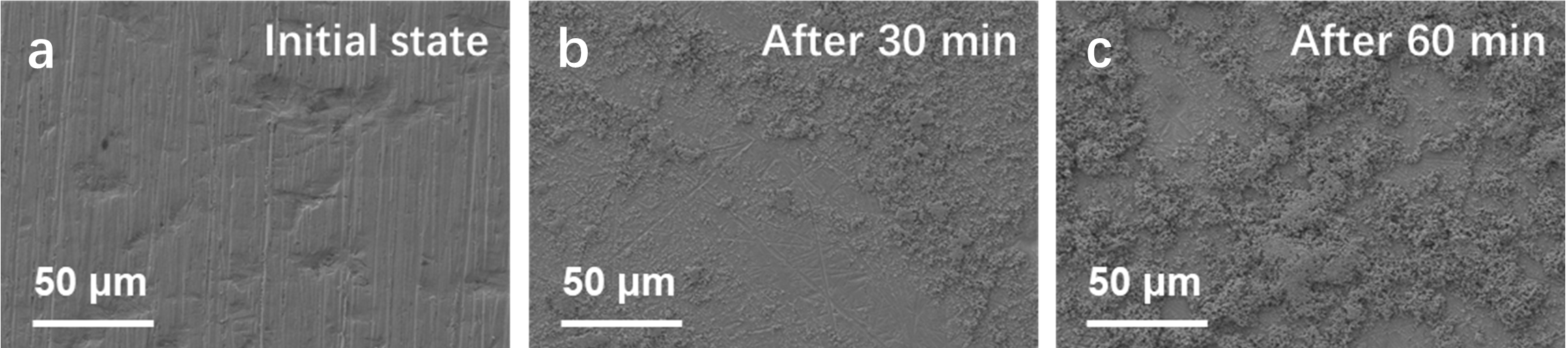


**Figure S12** (a-c) The SEM images of Zn anodes after cycling at 2 mA cm^-2^ for 0 min, 30 min and 60 min, respectively.


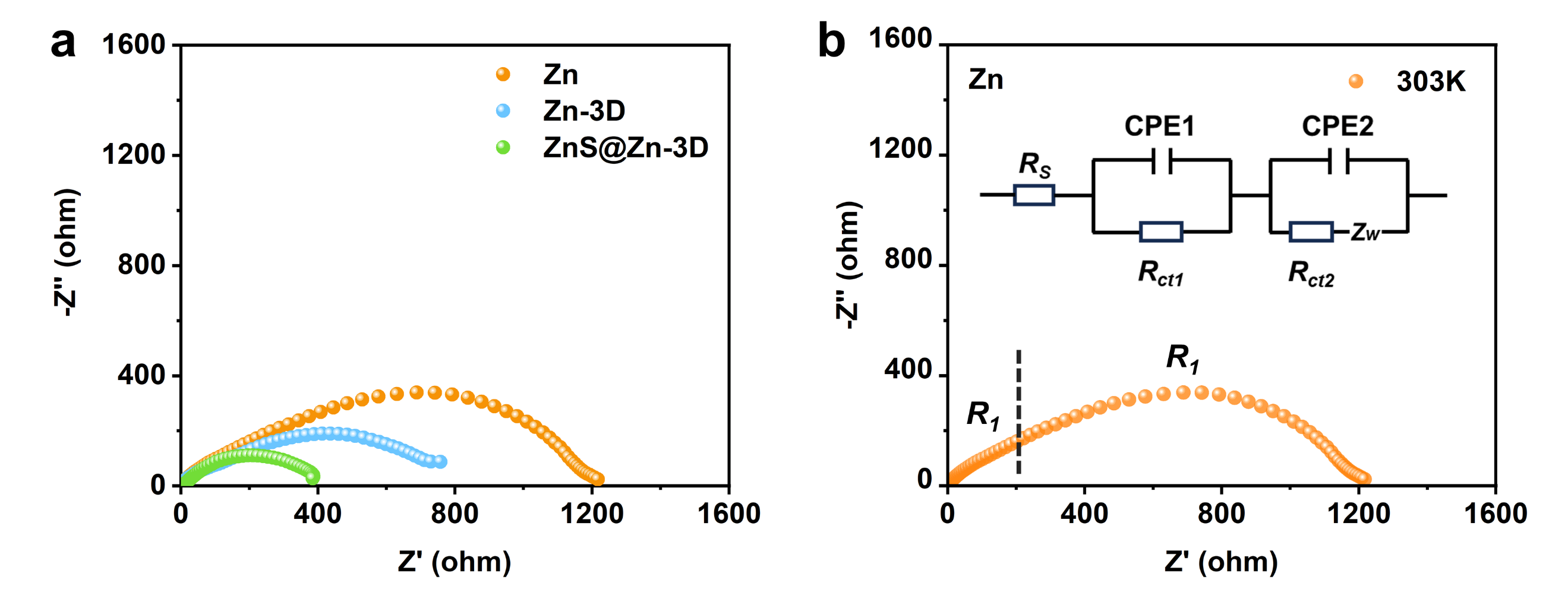


**Figure S13** The (a) Nyquist curves and (b) corresponding equivalent circuit diagrams of Zn, Zn-3D and ZnS@Zn-3D symmetric cells.





**Figure S14** Galvanostatic charge/discharge cycling voltage profiles of Zn, Zn-3D and ZnS@Zn-3D symmetrical cells at 0.5 mA cm^-2^ and 0.5 mAh cm^-2^.


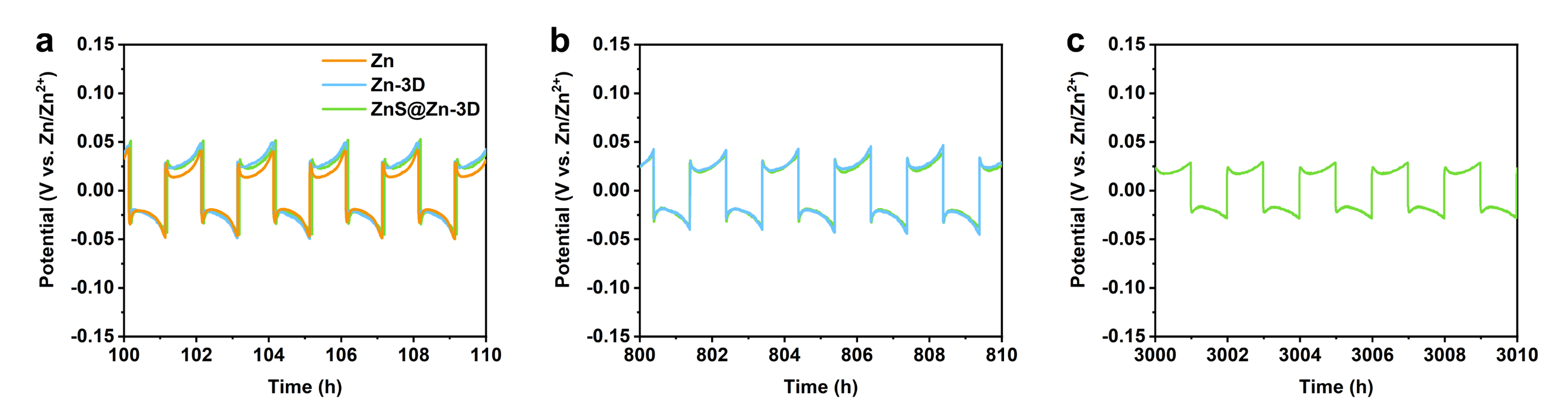


**Figure S15** (a-c) Galvanostatic charge/discharge cycling voltage profiles of Zn, Zn-3D and ZnS@Zn-3D symmetrical cells of at 0.5 mA cm^-2^ and 0.5 mAh cm^-2^.


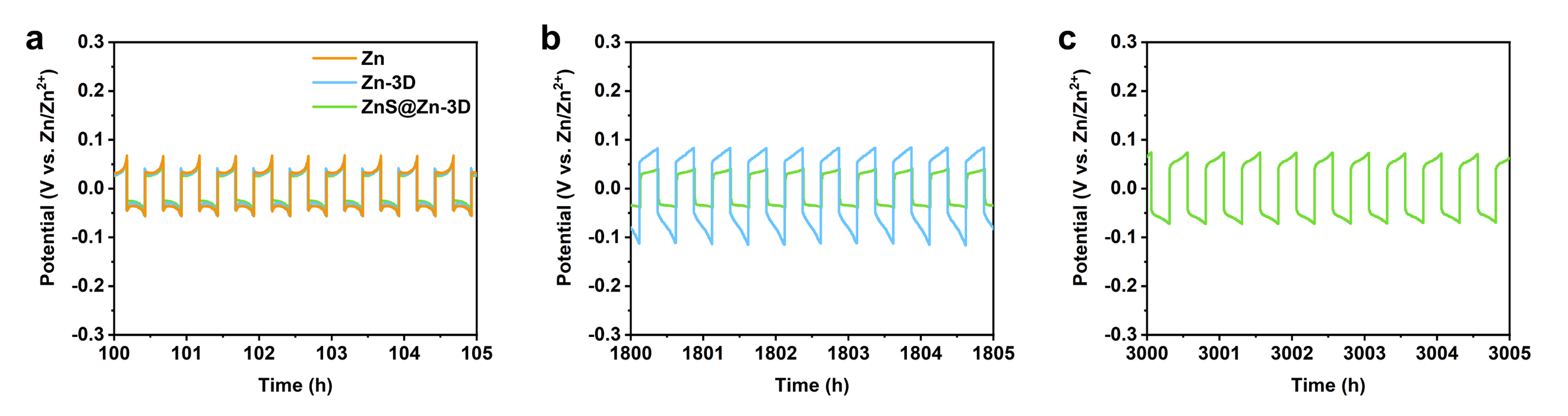


**Figure S16** (a-c) Galvanostatic charge/discharge cycling voltage profiles of Zn, Zn-3D and ZnS@Zn-3D symmetrical cells of at 4 mA cm^-2^ and 1 mAh cm^-2^.


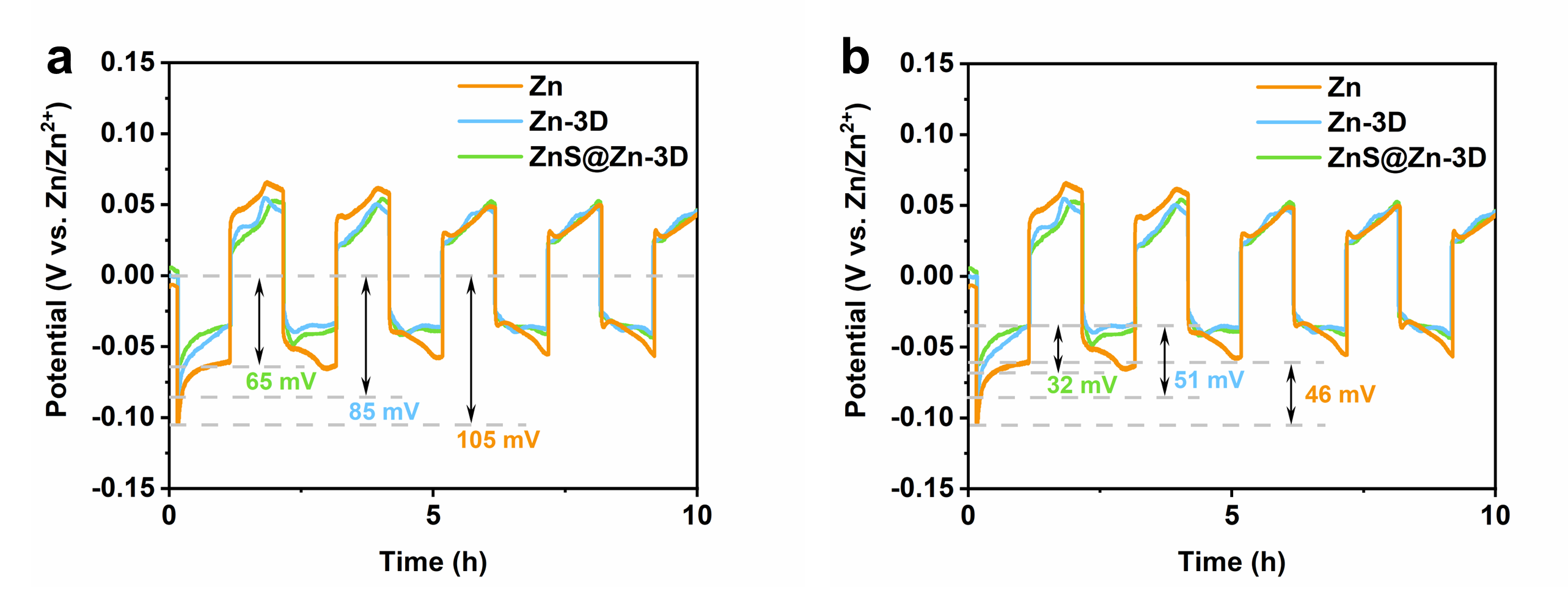


**Figure S17** (a, b) The nucleation voltage and nucleation overpotential of Zn, Zn-3D and ZnS@Zn-3D symmetric cells at 0.5 mA cm^-2^ and 0.5 mAh cm^-2^.


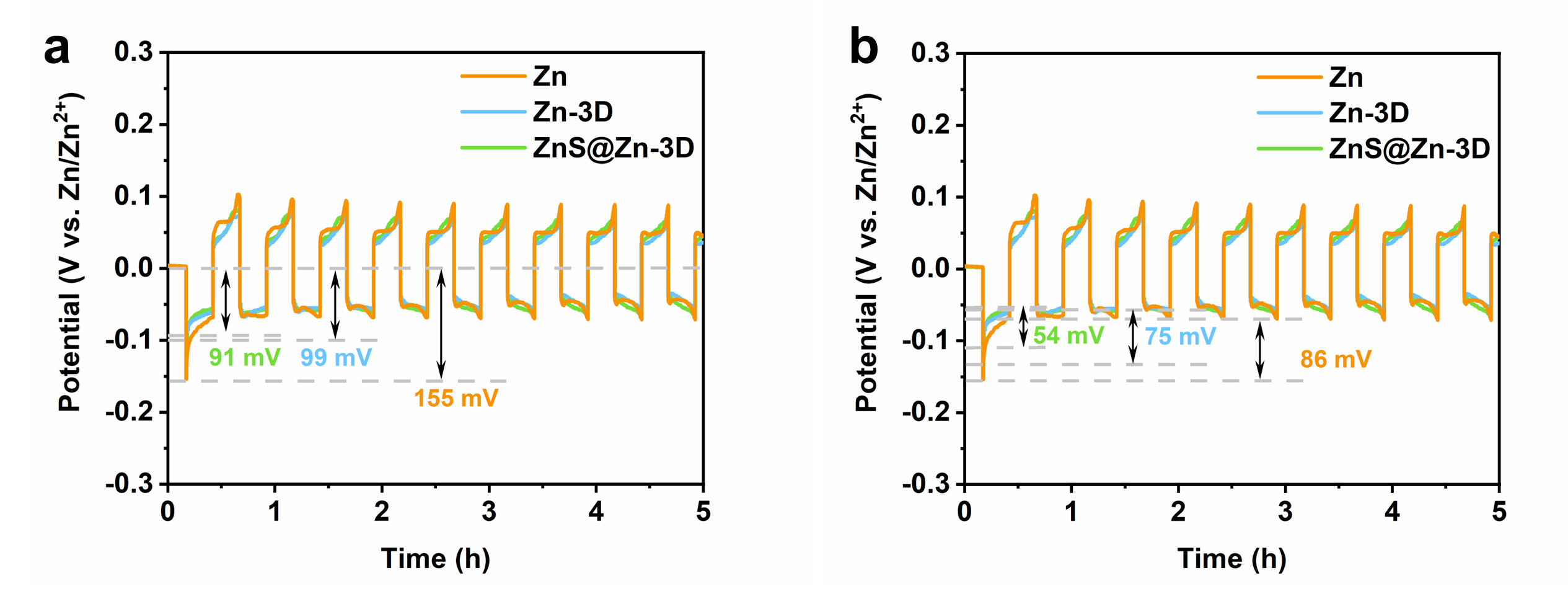


**Figure S18** (a, b) The nucleation voltage and nucleation overpotential of Zn, Zn-3D and ZnS@Zn-3D symmetric cells at 4 mA cm^-2^ and 1 mAh cm^-2^.


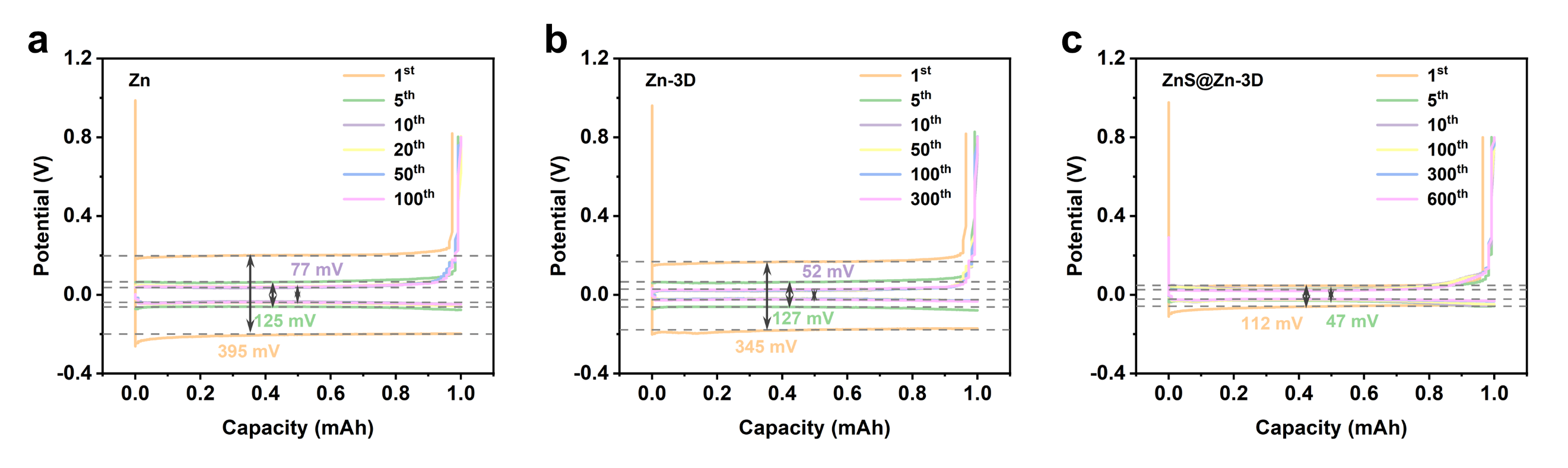


**Figure S19** The voltage-capacity curves of the Zn||Cu, Zn-3D||Cu, and ZnS@Zn-3D||Cu asymmetric cell at 2 mA cm^-2^ and 1 mAh cm^-2^.


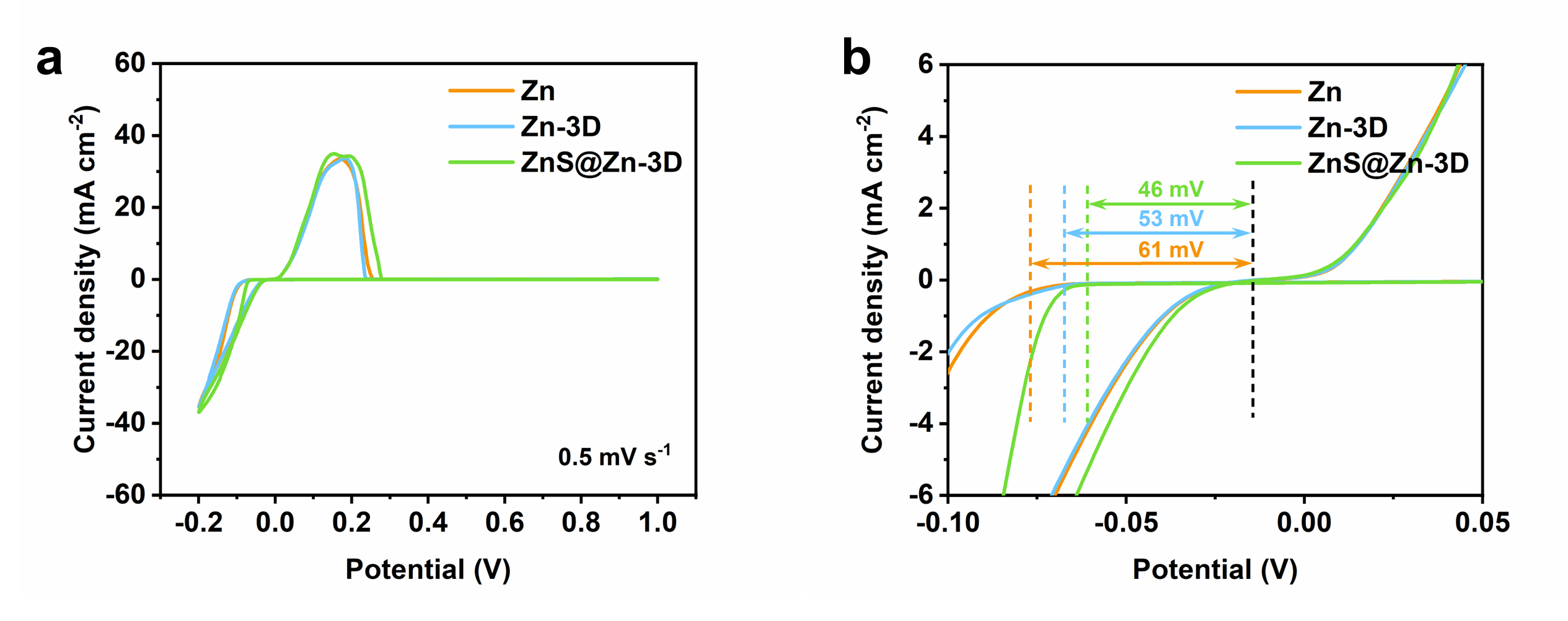


**Figure S20** (a, b) The CV curves of Zn||Cu, Zn-3D||Cu and ZnS@Zn-3D||Cu asymmetric batteries were at 0.5 mV s^-1^.


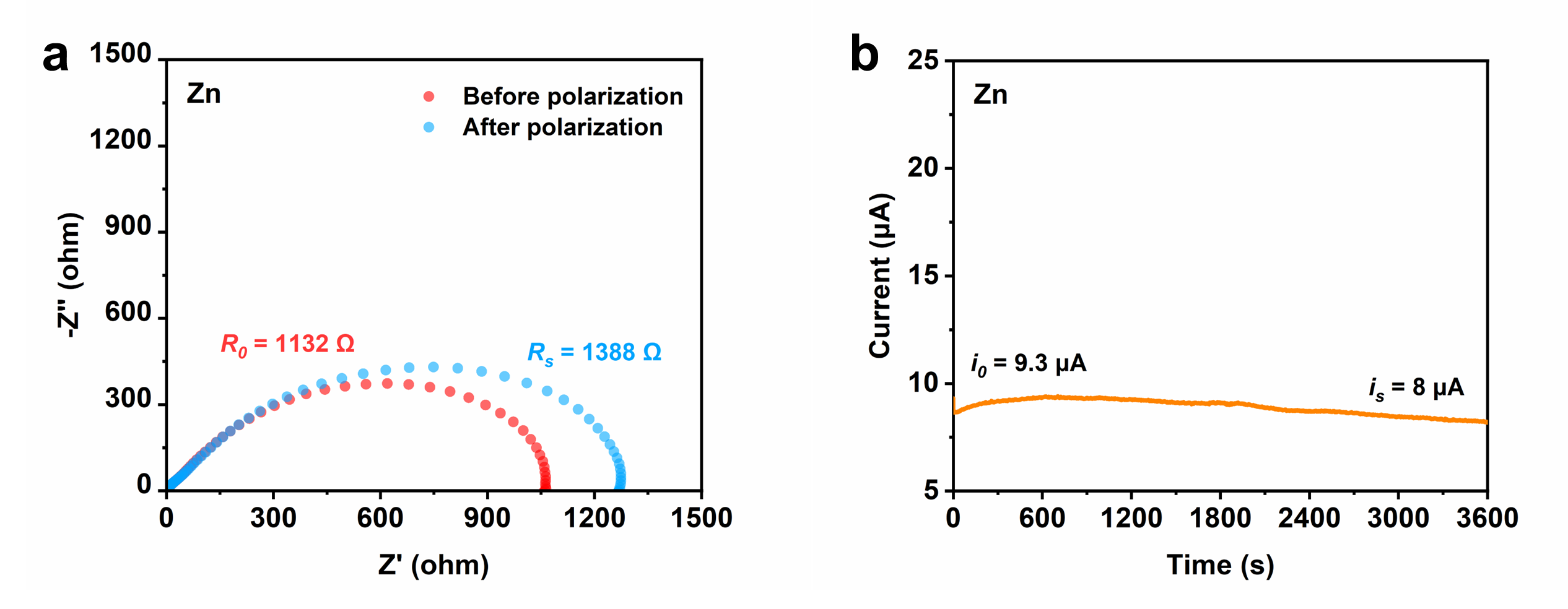


**Figure S21** (a) The Nyquist curve of Zn symmetric cells before and after polarization. (b) The chronoamperometry profiles at an overpotential of 10 mV.


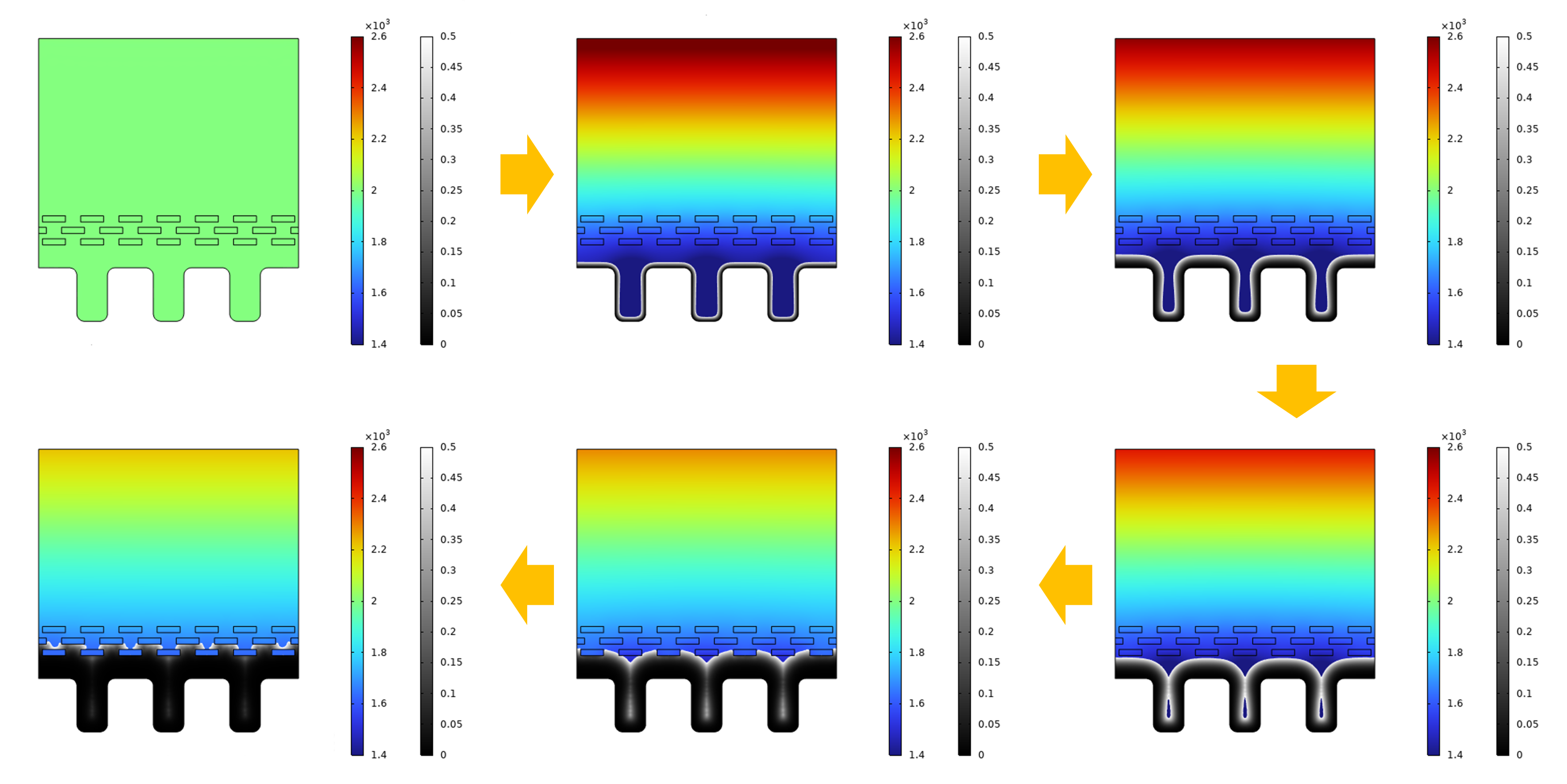


**Figure S22** The zinc deposition diagram of Zn-3D anode based on COMSOL finite element simulation.


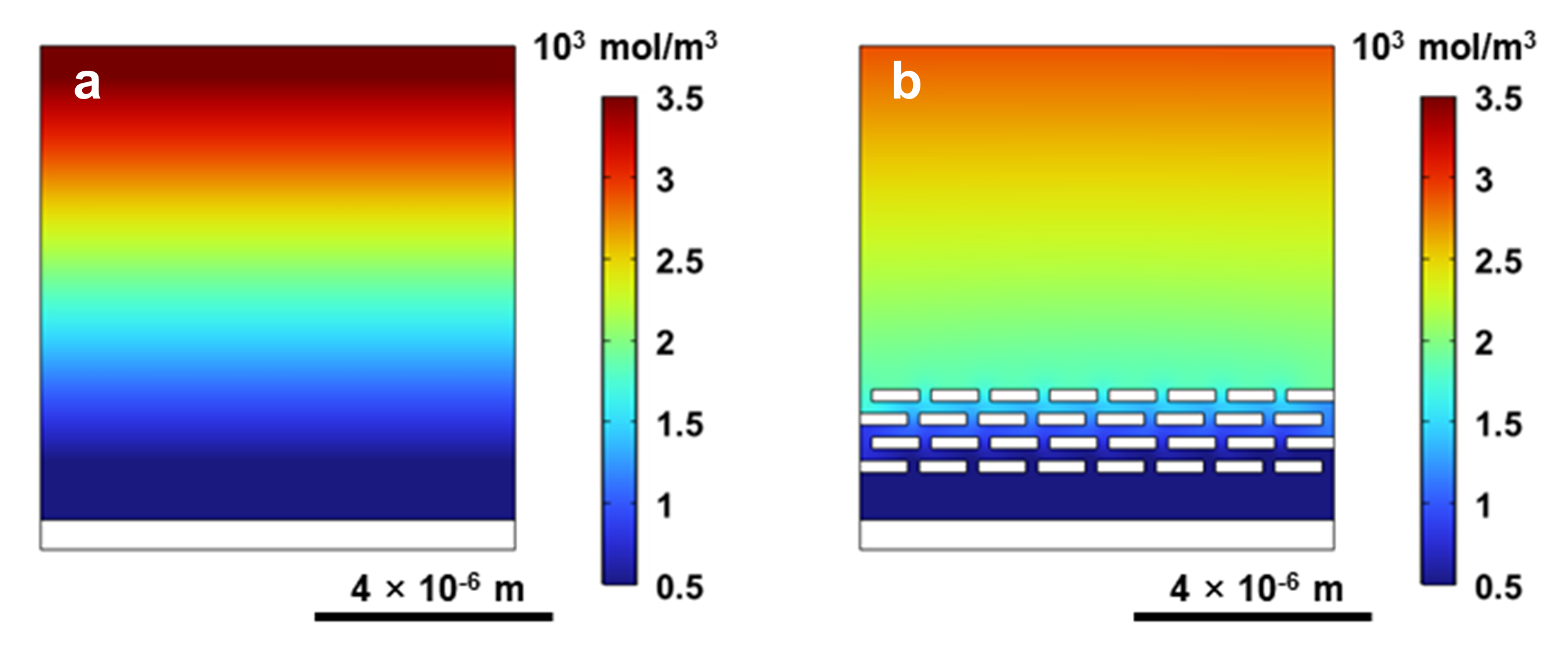


**Figure S23** The concentration distribution of Zn^2+^ ions on the surface of (a) Zn and (b) ZnS@Zn-3D anodes based on COMSOL finite element simulation.


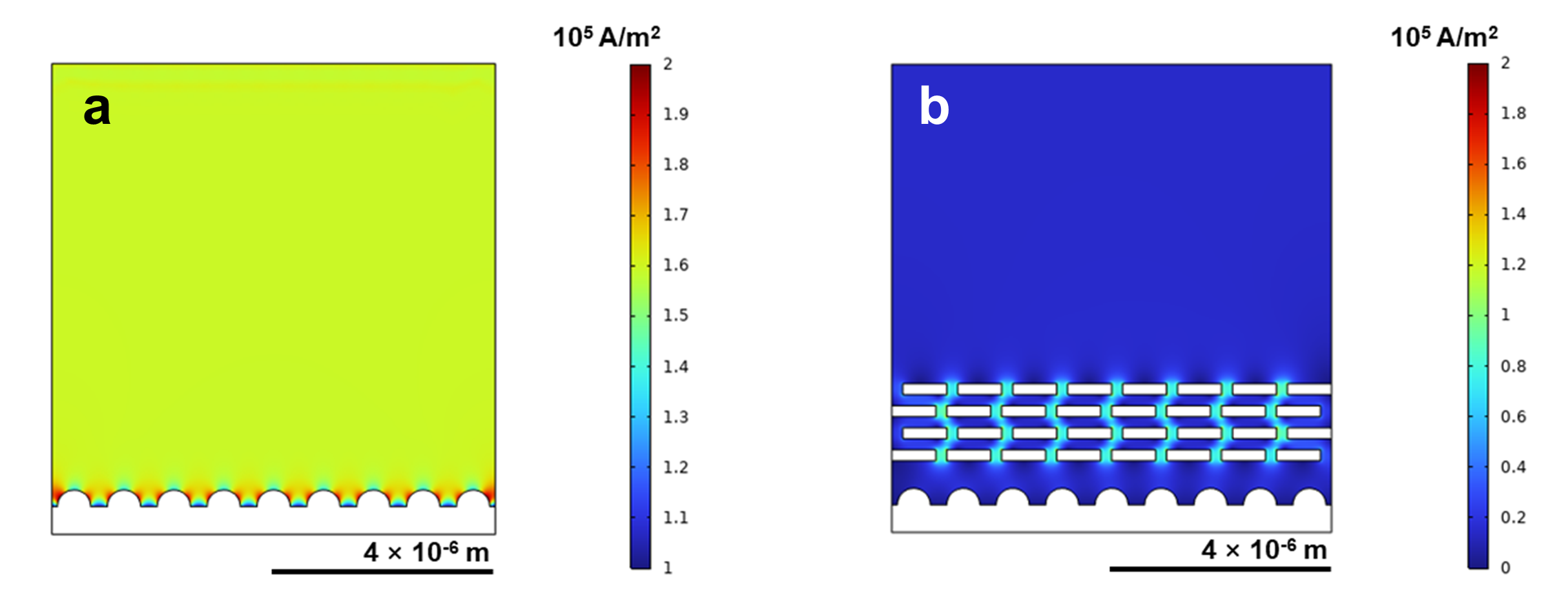


**Figure S24** The current density distribution on the surface of (a) Zn and (b) ZnS@Zn-3D anodes based on COMSOL finite element simulation.


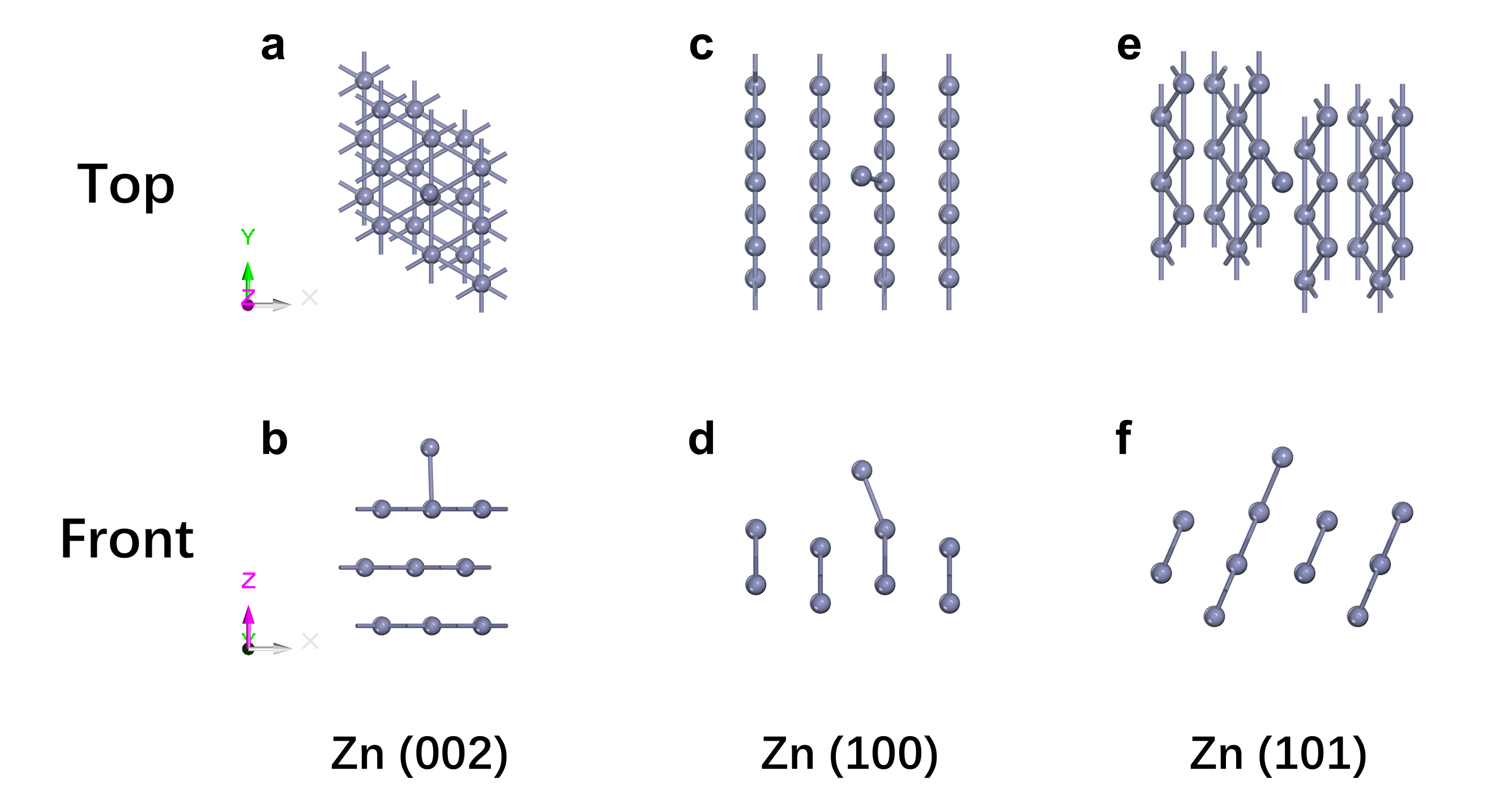


**Figure S25** Adsorption of Zn atoms on Zn crystal plane.


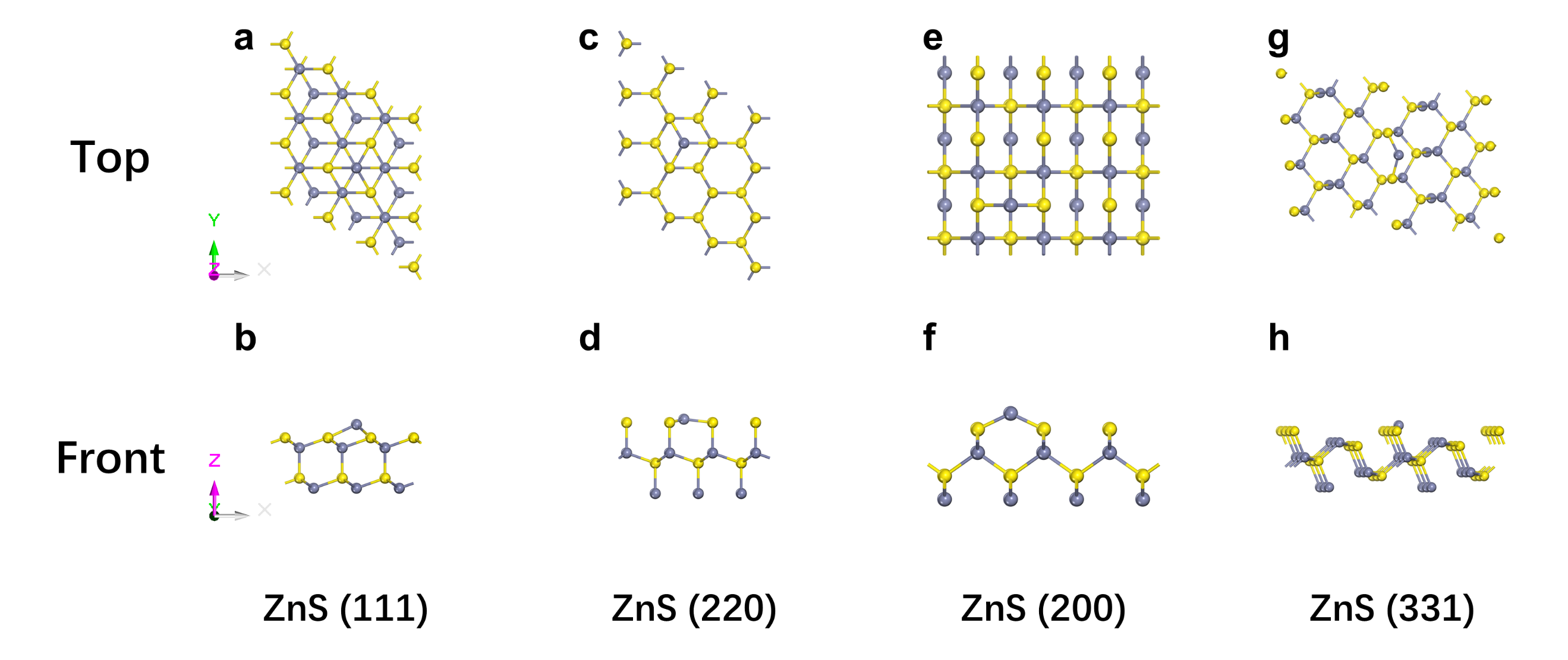


**Figure S26** Adsorption of Zn atoms on ZnS crystal plane.


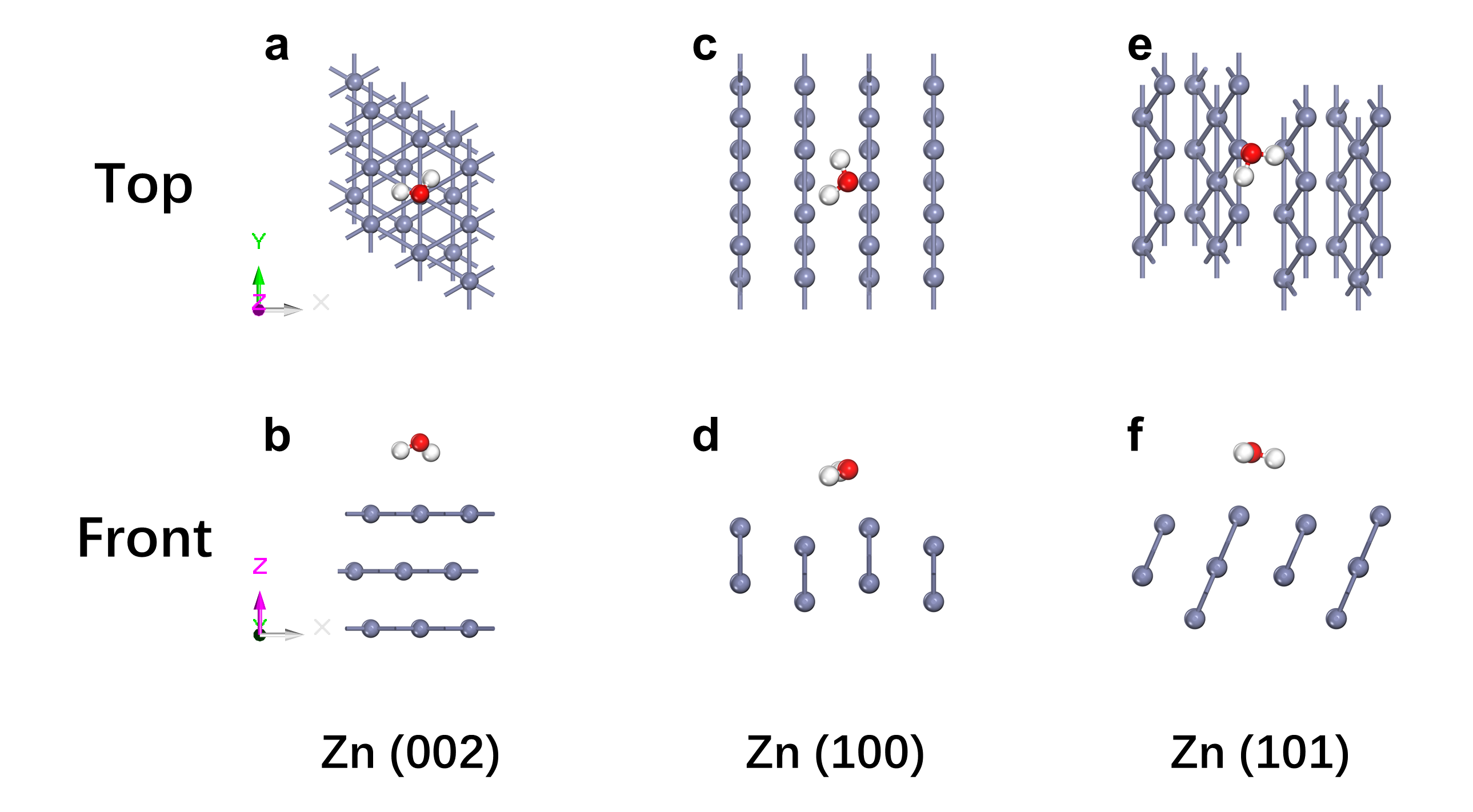


**Figure S27** Adsorption of H_2_O molecules on Zn crystal surface.


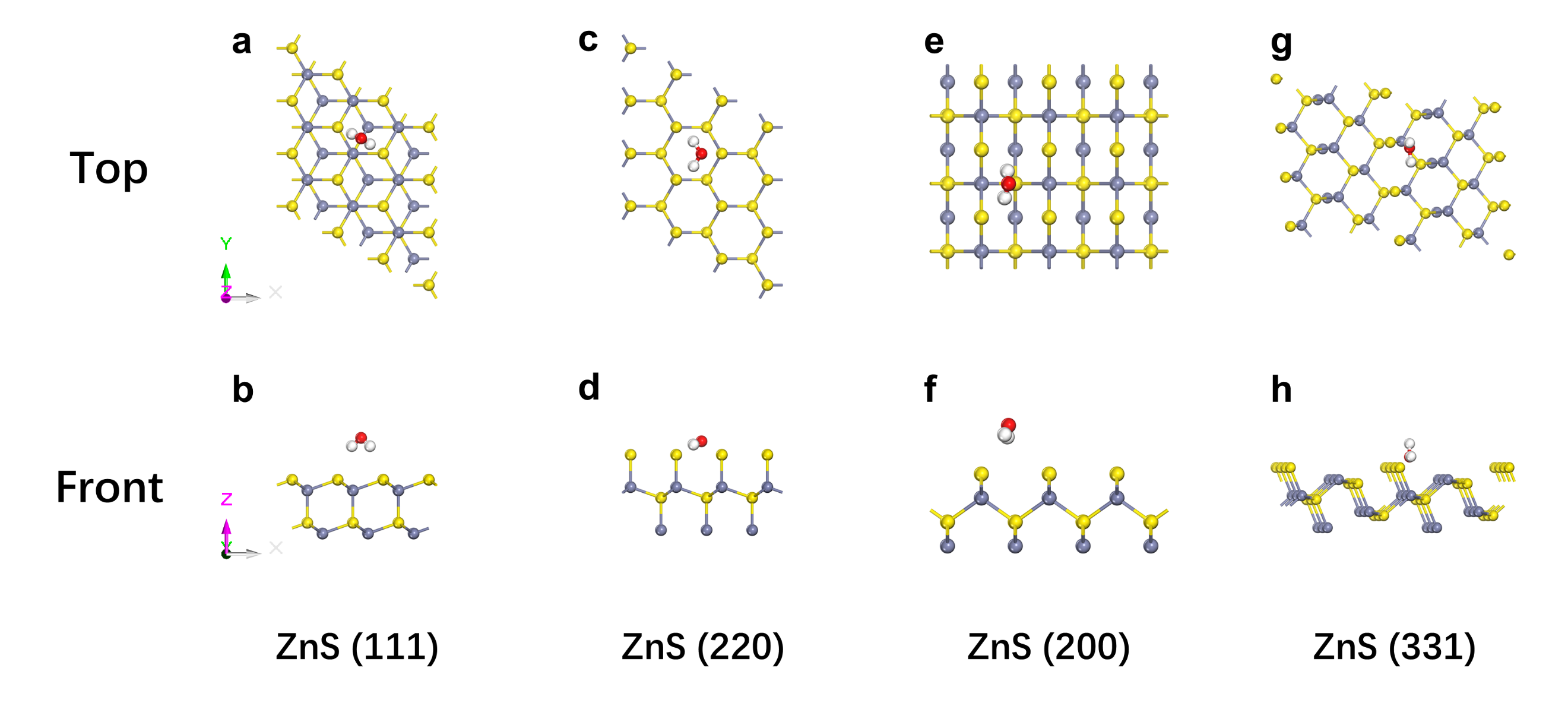


**Figure S28** Adsorption of H_2_O molecules on Zn crystal surface.


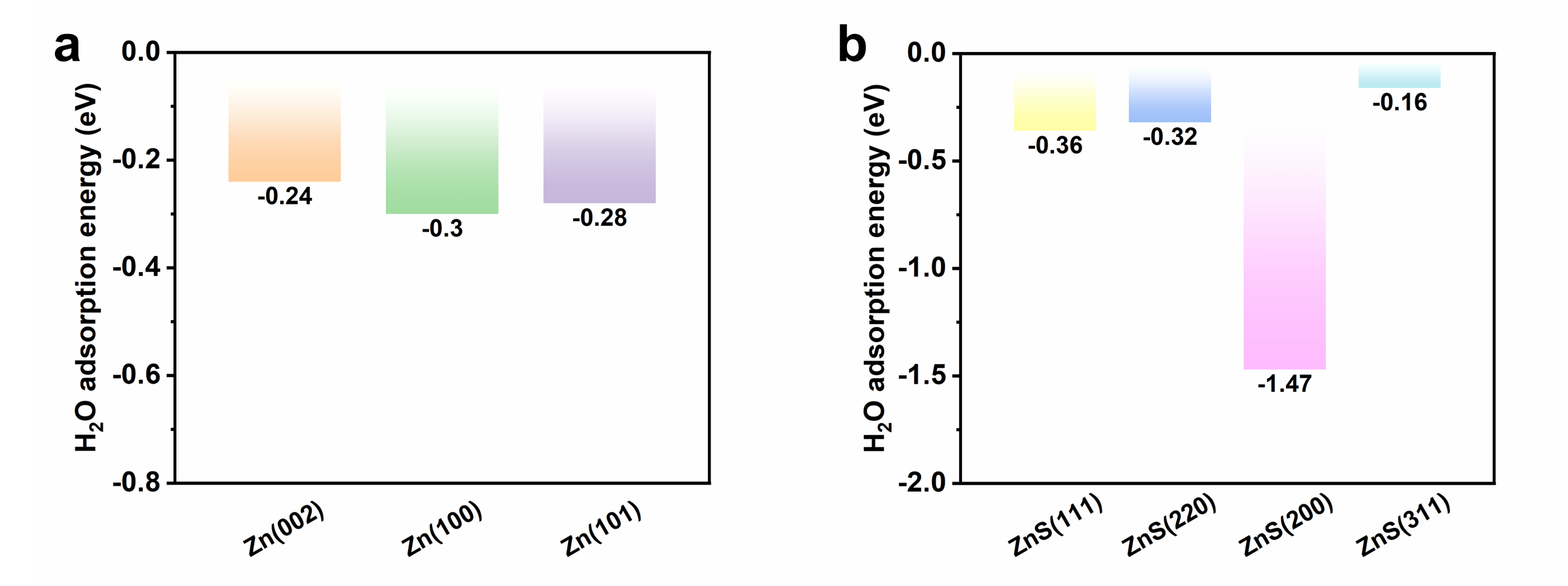


**Figure S29** The adsorption energy of H_2_O molecules on different crystal planes of Zn and ZnS.


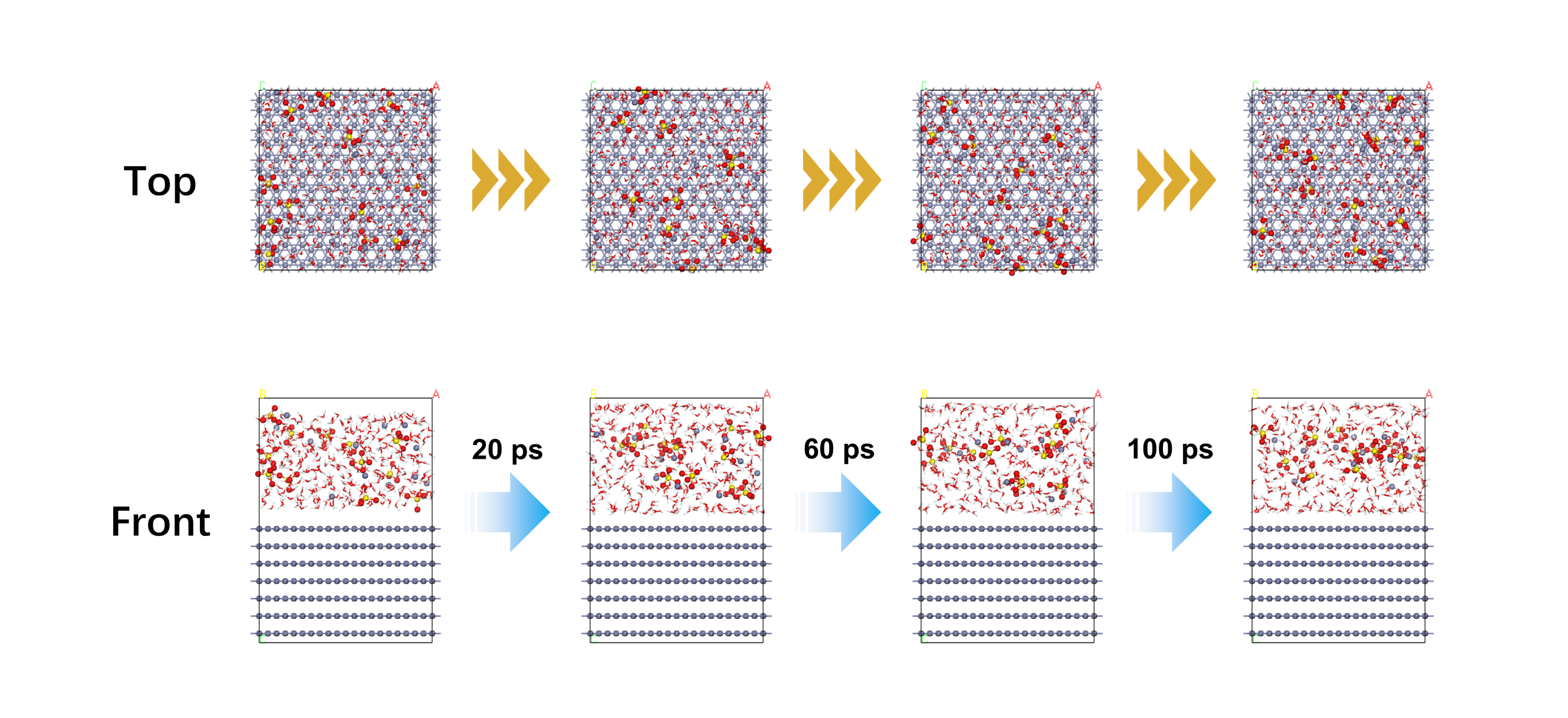


**Figure S30** The molecular dynamics simulation of 2M ZnSO_4_ on the surface of Zn anodes.


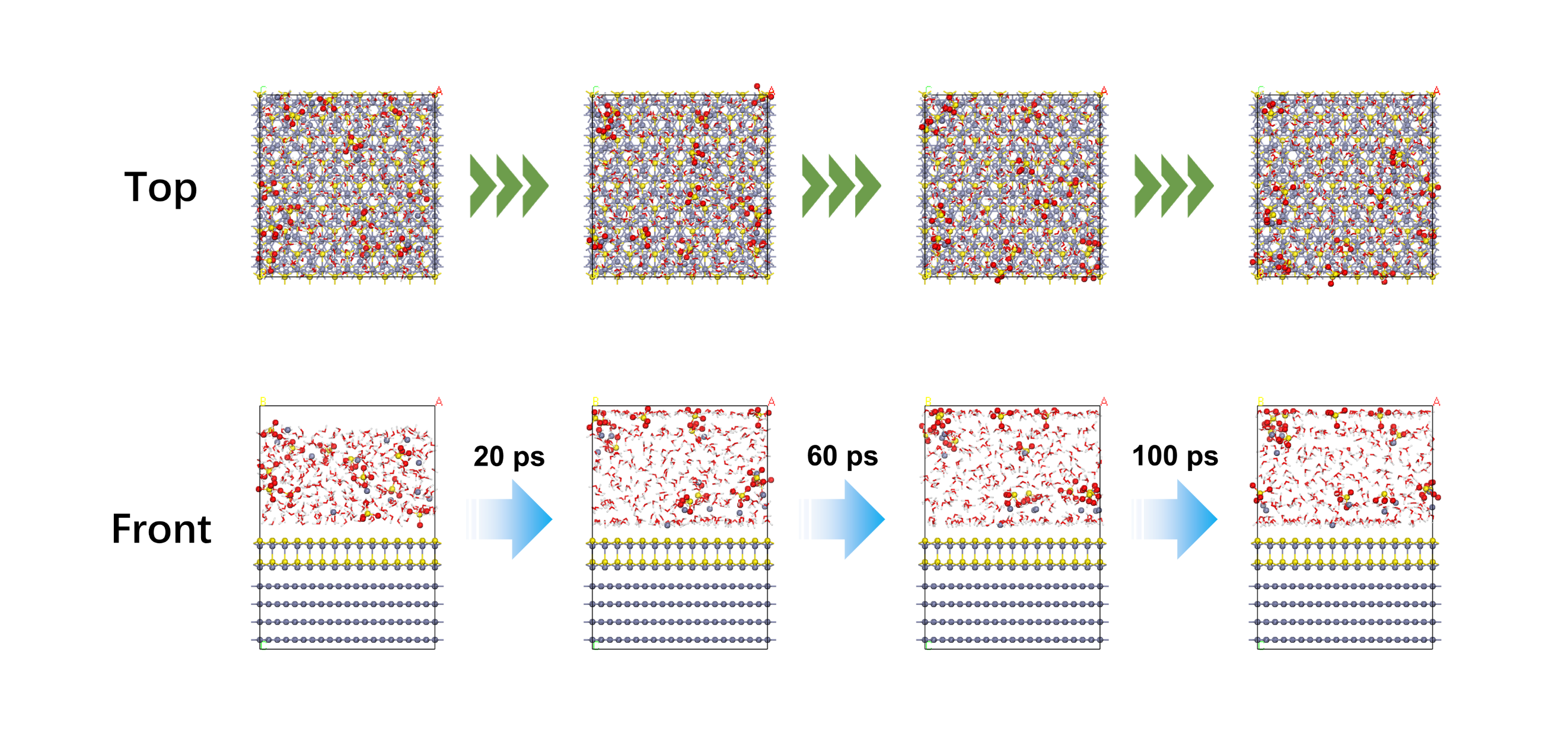


**Figure S31** The molecular dynamics simulation of 2M ZnSO_4_ on the surface of ZnS@Zn-3D anodes.


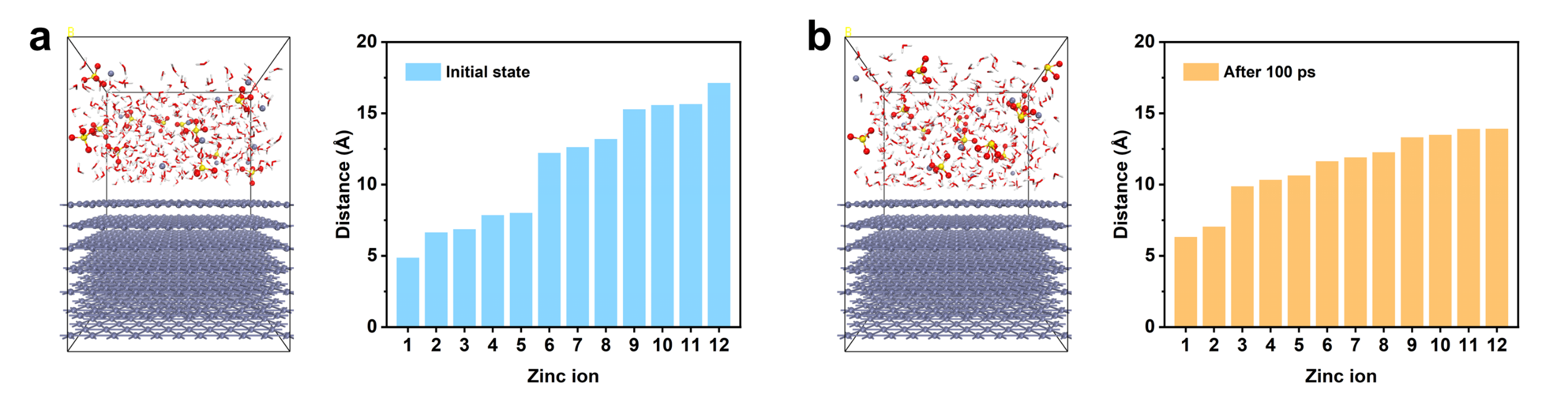


**Figure S32** The molecular dynamics simulation of Zn^2+^ ions on the surface of Zn anodes and the distance between Zn^2+^ ions and the surface: (a) initial state, (b) 100ps.


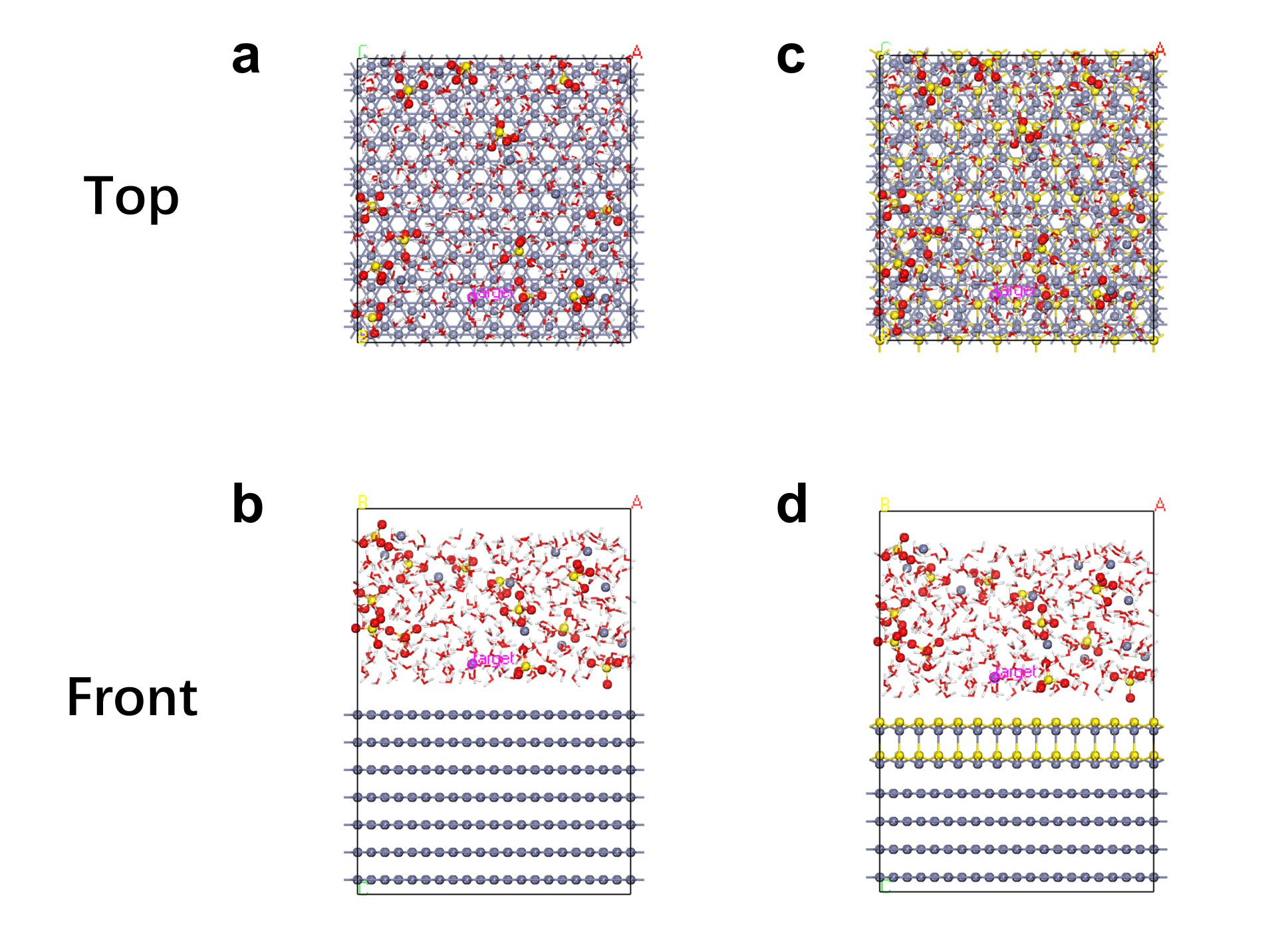


**Figure S33** The molecular dynamics test of the target Zn^2+^ (pink markings) at (a, b) the Zn and (c, d) ZnS@Zn-3D anode interface.


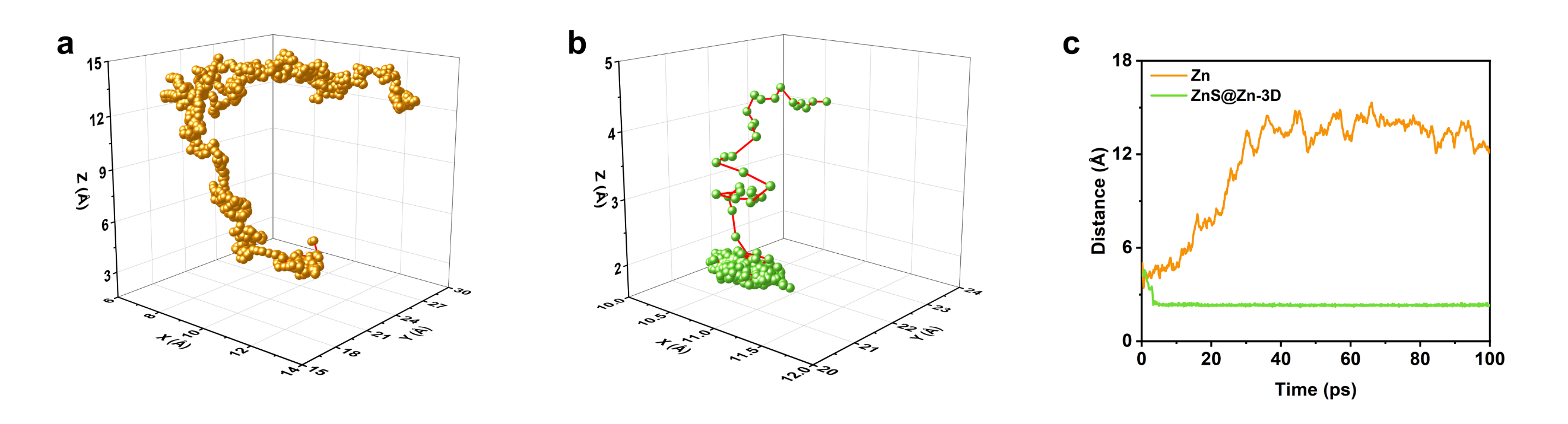


**Figure S34** The diffusion path of target Zn^2+^ at the interface of (a) Zn and (b) ZnS@Zn-3D anode and (c) the distance from the interface.





**Figure S35** The XRD spectra of the PC cathode.


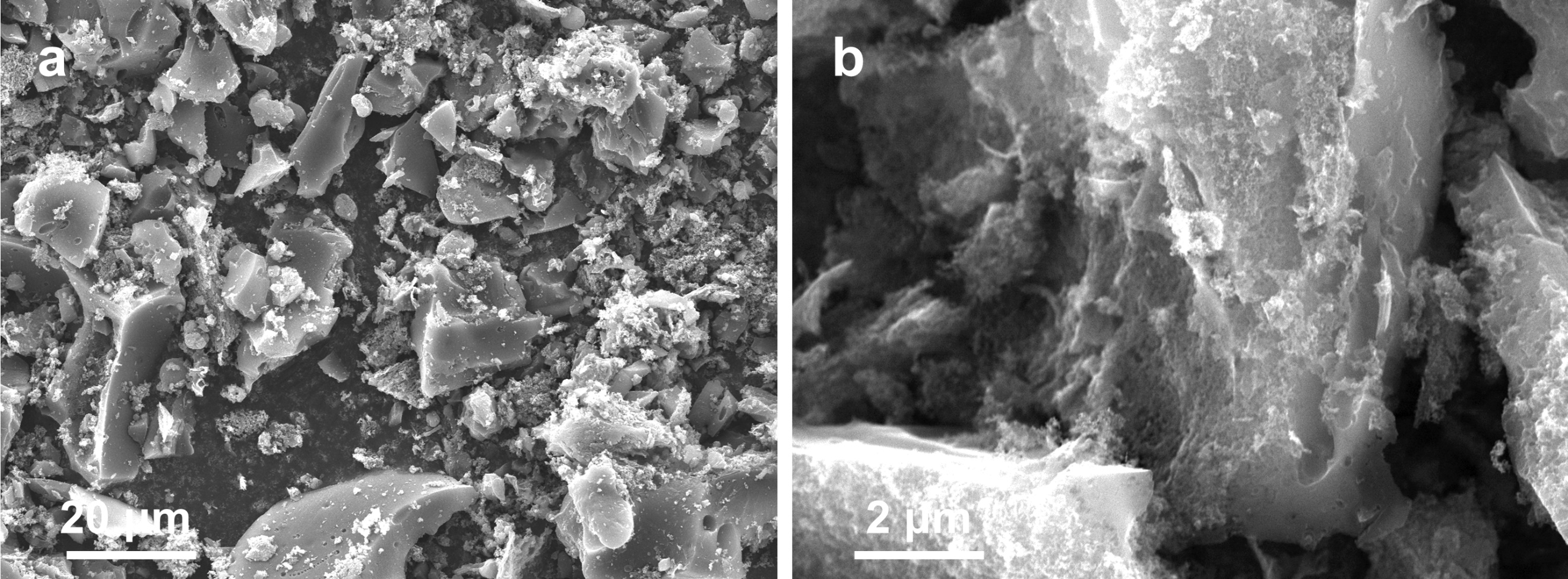


**Figure S36** SEM micro-morphology image of PC cathode.





**Figure S37** Slopes of impedance and low frequency of Zn//PC and ZnS@Zn-3D//PC full.





**Figure S38** The charge-discharge curves of Zn//PC full cells at different current densities.


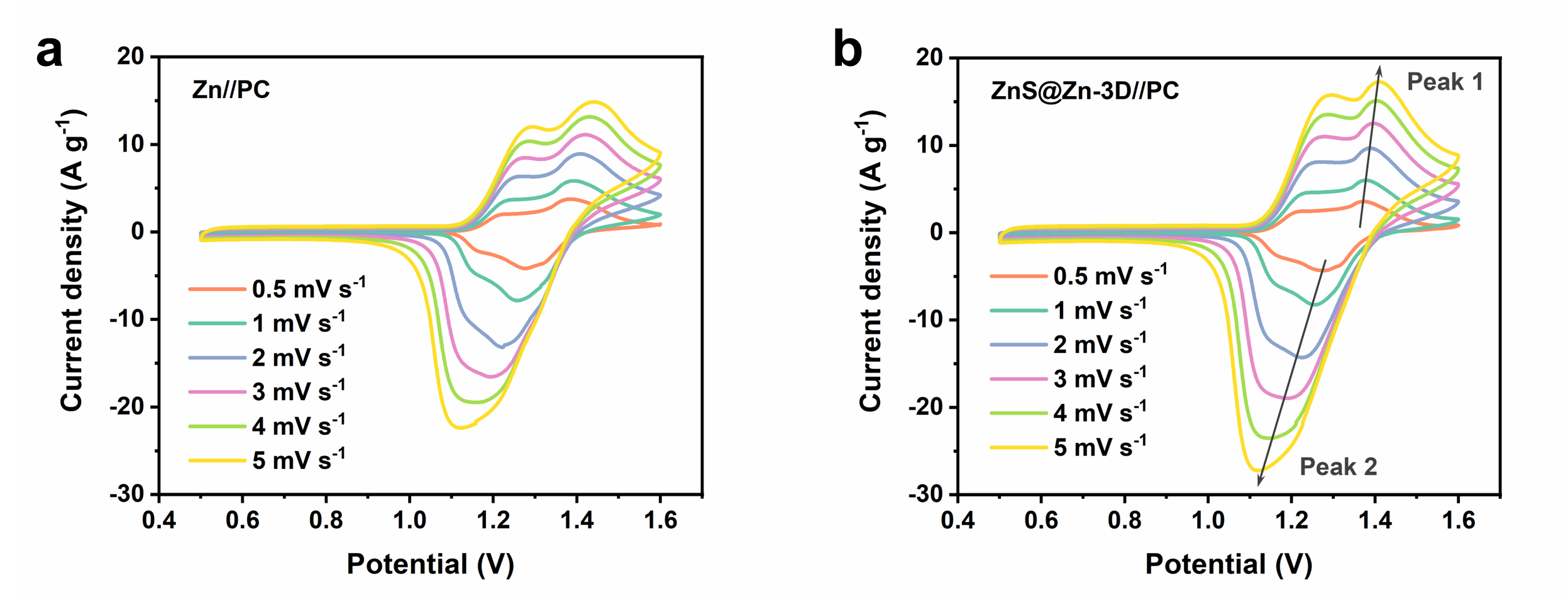


**Figure S39** The CV curves of (a) Zn//PC and (b) ZnS@Zn-3D//PC full cells are in the scanning rate range of 0.5-5 mV s^-1^.


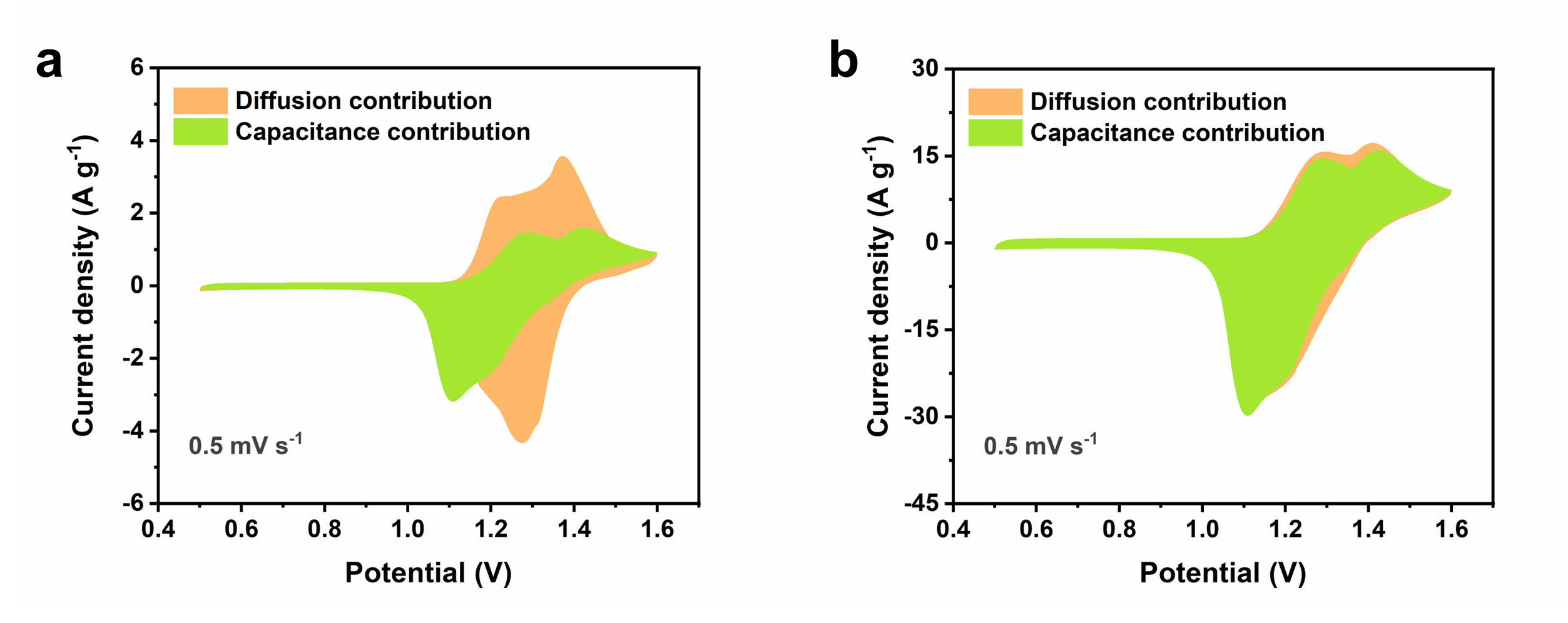


**Figure S40** The ratio of capacitance contribution and diffusion contribution of ZnS@Zn-3D//PC full cells at scan rates of (a) 0.5 mV s^-1^ and (b) 5 mV s^-1^, respectively.





**Figure S41** Long-term cycle stability test of ZnS@Zn-3D//PC full cells at a current density of 10 A g^-1^.

**Reference**

[1] D. Kundu, S. H. Vajargah, L. Wan, B. Adams, D. Prendergast, L. F. Nazar, *Energy Environ. Sci.* **2018**, 11, 881.

[2] J. Evans, C. A. Vincent, P. G. Bruce, *Polymer* **1987**, 28, 2324.
